# Photoinduced correlations in stochastic dynamics of a solid-state ionic conductor

**DOI:** 10.1038/s41467-026-72663-7

**Published:** 2026-05-15

**Authors:** Jackson McClellan, Alfred Zong, Kim H. Pham, Hanzhe Liu, Zachery W. B. Iton, Burak Guzelturk, Donald A. Walko, Haidan Wen, Scott K. Cushing, Michael W. Zuerch

**Affiliations:** 1 Department of Chemistry, University of California, Berkeley, CA USA; 2 Department of Chemistry and Biochemistry, The Ohio State University, Columbus, OH USA; 3 Materials Sciences Division, Lawrence Berkeley National Laboratory, Berkeley, CA USA; 4 Departments of Physics and of Applied Physics, Stanford University, Stanford, CA USA; 5 Division of Chemistry and Chemical Engineering, California Institute of Technology, Pasadena, CA USA; 6 Department of Chemistry, Purdue University, West Lafayette, IN USA; 7 Department of Applied Physics and Materials Science, California Institute of Technology, Pasadena, CA USA; 8 Advanced Photon Source, Argonne National Laboratory, Lemont, IL USA; 9 Materials Science Division, Argonne National Laboratory, Lemont, IL USA

**Keywords:** Optical spectroscopy, Ultrafast photonics, Chemical physics

## Abstract

Photoexcitation by ultrashort laser pulses plays a crucial role in controlling reaction pathways, creating nonequilibrium material properties, and probing complex molecular dynamics. The photoresponse following a laser pulse is generally nonidentical between exposures due to spatiotemporal fluctuations or the stochastic nature of dynamical pathways. However, most ultrafast pump-probe experiments struggle to distinguish intrinsic sample fluctuations from extrinsic apparatus noise, often missing deviations from the averaged response. Leveraging the stability and high photon flux of time-resolved X-ray micro-diffraction at a synchrotron, we characterized stochastic photoinduced dynamics in a solid-state ionic conductor. By analyzing temporal evolutions of the lattice parameter of a single grain, we found that shot-to-shot fluctuations are not independent. Instead, correlations exist between nonequilibrium lattice trajectories following adjacent shots, with a characteristic correlation length of approximately 1500 shots, corresponding to an energy barrier of 0.4 ± 0.1 eV, close to the activation energy of lithium-ion diffusion.

## Abstract (web summary)

This study employs time-resolved stroboscopic X-ray diffraction to reveal stochastic behavior of a solid-state ionic conductor. Through statistical analysis, the authors quantitatively determine the correlated transient lattice dynamics, which are linked to lithium ion motion.

## Introduction

Even though advances in laser technology have enabled the detection of ultrafast dynamics in biological systems^1^, quantum materials^2,3^, and chemical reactions^4^ down to the attosecond regime, probing stochastic processes that are intrinsic in these systems remains a challenge. In cases where fluctuations dominate the dynamics of interest, such as near the critical point of a phase transition or in a disordered medium riddled with topological defects, a conventional stroboscopic pump-probe scheme is ineffective as it averages out shot-to-shot variations in the pump-induced response. Even if the probe signal achieves a sufficiently high signal-to-noise ratio in a non-stroboscopic single-shot pump-probe measurement^5–7^, typically only the data point at one pump-probe delay is collected after a single pump shot, rendering it impossible to reconstruct the stochastic dynamics over time. Alternatively, a pump pulse may be followed by a train of probe pulselets via echelon-based optics^8–10^ to characterize the entire pump-induced evolution. However, the probe signal spread over individual pulselets can be too weak to yield useful information about the dynamics after only one pump shot, and extensive averaging over multiple pump shots is often necessary. Fundamentally, there is an upper limit on how strong the probe pulse (or pulselets) can be so that it does not significantly alter the dynamics under investigation. This limit hence imposes a trade-off between the signal-to-noise ratio and how many delay times can be measured, preventing access to the full stochastic dynamics that differ from one pump shot to another.

Here, we tackle this challenge in studying stochastic dynamics via a statistical approach to observe correlations between random events, which reveal underlying physical properties of the material system. Although similar statistical analyses have been performed on dynamic chemical and biological flow systems^11–13^, our method focuses on the dynamic behavior of an individual grain of a polycrystal and employs a stroboscopic data acquisition scheme as used in non-single-shot pump-probe experiments. Due to the necessity of repeated data acquisitions, one requirement of this method is the use of a stable probe source and high measurement statistics to mitigate the averaging over several pulse events. A key to this method relies on the low noise level associated with the data collection process that can be plagued by instrumental instability or low photon counts in table-top ultrafast laser-based measurements. In our experiment, we overcome these problems by utilizing the stability and high photon flux of a synchrotron-based time-resolved X-ray setup such that the experimentally observed signal variation is dominated by the intrinsic sample fluctuations in the photoinduced response^14–17^. This method, with careful consideration of measurement parameters like repetition rate and data sampling, can be used to further characterize metastable states related to lattice, charge, spin, and orbital degrees of freedom, where quasi-equilibrium conditions result in a non-uniform response from a light pulse^18–23^. We term this method *nonequilibrium noise correlation spectroscopy*, which can be applicable to time-resolved experiments where stochastic processes are prevalent.

**Fig. 1:**
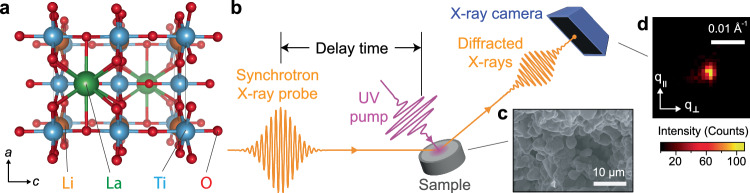
Overview of the powder sample and the pump-probe setup. **a** Crystal structure of the tetragonal LLTO in equilibrium. Graphics rendered by VESTA^74^. **b** Schematic of the experimental setup for synchrotron-based time-resolved hard X-ray micro-diffraction (see “Methods”). **c** Scanning electron micrograph of the powder LLTO sample pressed into a pellet, which was used in the time-resolved X-ray experiment. **d** Raw diffraction image of the (004) Bragg peak of a particular crystalline grain recorded before pump pulse arrival. **q**_⊥_ is along the *c*-axis of the grain, while **q**_∥_ lies in the *a*-*b* plane of the grain.

To demonstrate this capability of unraveling dynamical correlations in the transient stochastic response, we investigate Li_0.5_La_0.5_TiO_3_ (LLTO), a highly conductive solid-state electrolyte candidate for lithium ion batteries^24–27^. LLTO has a perovskite crystal structure (Fig. 1a) with alternating vacancy-rich and poor layers^28^, where the low diffusion barrier leads to high lithium ion conductivity^29^. The mobility of lithium ions via LLTO is dependent on the crystal structure^30^, which changes substantially upon the insertion and extraction of lithium ions as observed in X-ray diffraction^29^. Conversely, if the crystal structure changes upon photoexcitation due to nonthermal phonon population^31,32^ and transient heat deposition^33,34^, one also expects varying dynamics from shot to shot due to the stochastic location of the lithium ions in the crystal, which are free to migrate either due to thermal activation^35^ or photoexcitation^36^. Therefore, the mobile lithium ions in LLTO offer the ideal platform to investigate stochastic fluctuations in lattice dynamics following photoexcitation, which can in turn yield insight into the strong coupling between lithium ion diffusion and the crystalline lattice^30,37^ to design next-generation solid-state electrolytes.

To this end, we monitor the evolution of the lattice parameters of LLTO following above-bandgap photoexcitation using synchrotron-based time-resolved X-ray diffraction (see “Methods” for details). Such excitation in LLTO is expected to cause heat-induced initial lattice expansion and subsequent relaxation that modulate lithium site occupations because it is known that the activation energy of ionic transport is closely tied to lattice distortions^37^. In this work, we uncover a hidden correlation between lattice motions following neighboring pump shots with a characteristic “correlation length” of approximately 1500 laser shots, which corresponds to a ~0.4 eV energy barrier for lithium-ion diffusion. The framework of nonequilibrium noise correlation spectroscopy introduced in this work provides a new understanding of the potential role of photoinduced structural change in the lithium-ion conduction process, opening the avenue for harnessing tailored laser pulses for manipulating ionic conduction in solids.

## Results

### Time-resolved X-ray micro-diffraction

A schematic of the setup is shown in Fig. 1b (see “Methods” for a more detailed description). As industrial-scale solid-state ionic conductors are often synthesized as sintered pressed powder pellets^38^, the LLTO sample under investigation was prepared in a similar polycrystalline form, whose typical grain size can be up to a few micrometers (Fig. 1c). The high momentum-resolution of the setup as well as the comparable X-ray beam spot size to the LLTO grain size make it possible to observe individual Bragg peaks instead of Debye-Scherrer rings (Fig. 1d). This enables us to focus on the stochastic lattice dynamics of a single grain without an averaging effect over the whole powder ensemble.

**Fig. 2:**
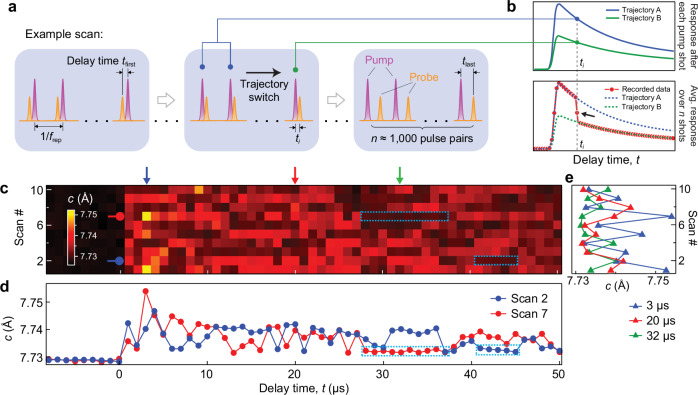
Stochastic photoinduced lattice response in LLTO. **a** Schematic of the stroboscopic pump-probe measurement using a pump laser repetition rate of *f*_rep_ = 1 kHz. At each pump-probe delay time *t*_*i *= 1,…,59_, the system response is integrated over *n* ≈ 1000. In the measurement, the pump-probe delays are changed electronically in a sequential manner, as indicated by the gray arrows. A scan consists of a complete cycle of delays *t*_1_, …, *t*_59_ (−8 to 50 μs), and it is repeated multiple times with a negligible time overhead in between successive scans; here, only one particular scan is drawn. **b** *Upper panel:* Schematic of stochastic lattice expansion trajectories induced by selected pump pulses, where the color-coded dots indicate the data points probed by the X-ray pulse at a particular time delay *t*_*i*_ in a scan. Only two out of many possible trajectories are shown as examples. *Lower panel:* Schematic of recorded data for a particular scan, where a discontinuity (highlighted by the black arrow) occurs due to a stochastic change of the photoinduced lattice trajectory during data collection at delay time *t*_*i*_. Only one such discontinuity is shown as an example, but many such discontinuities can occur during the course of one scan. **c** Measured time evolution of the *c* lattice parameter of a particular LLTO crystalline grain extracted from the (004) Bragg peak, shown for 10 consecutive scans under a 349-nm pump laser with a 67 mJ/cm^2^ incident fluence at room temperature. **d** Two representative time traces in scan 2 and scan 7 taken from panel (**c**), showing nearly identical values before *t* = 0 but exhibiting dichotomous light-induced responses due to the stochastic nature of nonequilibrium lattice dynamics. Dashed boxes in (**c**, **d**) highlight examples of a streak of similar *c* values before or after a discontinuous shift in value. **e** Three vertical line cuts selected from panel (**c**) (see color-coded arrows), demonstrating the variation of the *c*-axis parameter across different scans at the same pump-probe delay times.

To understand how the experiment is sensitive to shot-to-shot variation of the lattice response despite a conventional stroboscopic pump-probe scheme, it is worth revisiting the measurement protocol, summarized in Fig. 2a. The repetition rate of the ultraviolet pump laser was 1 kHz, and the X-ray diffraction peak was measured at each pump-probe delay time ranging from −8 to 50 μs in 1 μs steps (*t*_*i *= 1,…,59_). Diffraction intensities of *n* pump-probe pulse pairs were averaged for each *t*_*i*_ for 1 s, meaning that each pixel in Fig. 2c is an average of *n* ≈ 1000 pulse pairs. Upon the completion of one full pump-probe delay scan, we repeated the procedure for a total of *N* scans (*N* = 10) with nearly zero waiting time between successive scans. In general, the sample response after every single pump laser pulse can follow different trajectories due to fluctuations (Fig. 2b, upper panel). Even though we only captured a single point out of the entire photoinduced evolution at each delay time *t*_*i*_ (solid dots in the upper panel of Fig. 2b), as illustrated in the lower panel of Fig. 2b, we can still detect the stochastic variation between certain shots based on abrupt discontinuities in the recorded time trace (highlighted by the black arrow), especially if such discontinuities occur long after the region of pump-probe temporal overlap and if they are much larger than the noise level of the measurements (see Supplementary Note 4 for noise estimates).

**Fig. 3:**
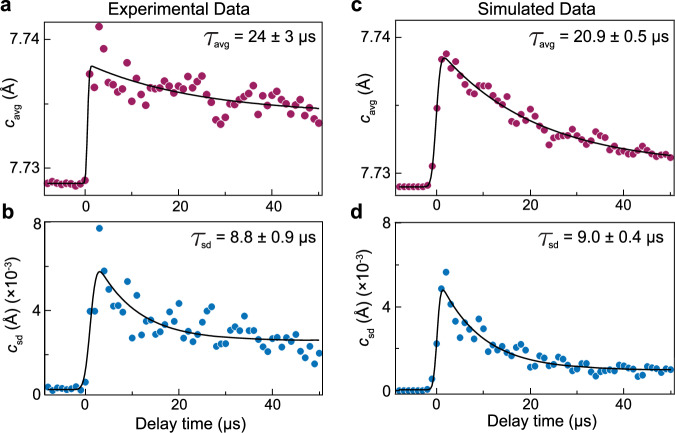
Average lattice response and its fluctuations. **a** Time evolution of the *c* lattice parameter averaged over the 10 scans in Fig. 2c. **b** Time evolution of the standard deviation *c*_sd_ derived from the 10 scans in Fig. 2c. In (**a**, **b**) the solid curves are fits to a phenomenological model in Eq. (1). The characteristic relaxation time *τ*_sd_ is markedly shorter than *τ*_avg_. **c**, **d** Simulated data corresponding to the measured results in panels (**a**, **b**), reproducing the difference between *τ*_avg_ and *τ*_sd_. See the main text and Supplementary Note 6 for simulation details.

These discontinuities are clearly observed in our measurements. Figure 2c shows the *c*-axis lattice parameter as a function of pump-probe delay time for ten scans, two of which are shown in Fig. 2d (see Supplementary Note 1 for the extraction of *c* from diffraction images). Upon photoexcitation, on average, the lattice exhibits a sudden *c*-axis expansion of more than 0.1% followed by a slow recovery over tens of microseconds (Fig. 3a). For individual scans, however, the *c* value experiences seemingly random discontinuities in the time trace after pump pulse arrival. By contrast, the variation of *c* before photoexcitation is much smaller (e.g., 25-times smaller in scan 7 in Fig. 2d; see Supplementary Note 4 for noise estimates). This dichotomy of the noise level before and after *t* = 0 excludes extrinsic measurement uncertainties due to factors such as instability of the X-ray beam, which is expected to yield a similar noise level at all time delays.

Another extrinsic factor that must be addressed for causing discontinuities of the *c* lattice constant is fluctuations in the pump laser pulse energy, where a large fluctuation of the laser pulse energy from one pulse to another can, in principle, lead to stochastic sample responses as well. To rule out any contribution from the pump laser, a comparative analysis between the pump laser power fluctuations and those observed in LLTO was performed in Supplementary Note 4. We calculated the coefficient of variation (defined as the standard deviation divided by the average of a time series of data) for the changes in the LLTO *c* lattice parameter after time zero and for a recorded time trace of the pump laser power at beamline 7ID-C. If changes in the pump laser power directly resulted in different values of the *c* parameter, the coefficient of variation would be similar across *c* lattice scans and the pump power trace. However, we see that the *c* trace has a coefficient of variation which is more than an order of magnitude larger than any recorded pump laser fluctuations, suggesting that the noise of the pump laser is unlikely to account for the observed stochastic response in LLTO. Furthermore, to rule out the scenario where a tiny deviation of the pump laser energy may be nonlinearly amplified to a large change of the *c* lattice constant, we recorded the *c* lattice displacement as a function of pump laser power in Supplementary Fig. 6c. Each point in Supplementary Fig. 6c represents the change in the *c* lattice parameter under illumination by the pump laser compared to its value in the dark, plotted as a function of varying pump laser fluence. This plot reveals a linear trend in the lattice constant as a function of pump power, hence excluding the possibility of nonlinear changes of the lattice parameter due to small fluctuations of the pump laser pulse. Most importantly, previous stroboscopic pump-probe measurements from the same experimental setup under similar conditions have not observed this effect^39^ (see Supplementary Note 3 for a statistical analysis of the photoinduced lattice response of Pb(Zr_0.2_Ti_0.8_)O_3_). Standard samples, which are not expected to exhibit stochastic responses, do not show this behavior^39–41^. In LLTO, mobile lithium ions and varying strain conditions are expected to modulate transient lattice conditions between successive pump laser shots, making it plausible that the observed phenomenon is caused by trajectory switching of the *c* parameter in LLTO.

Upon closer scrutiny, two features of the stochastic response reflected in these discontinuities stand out in Fig. 2c–e. First, the relative magnitude of a shift in *c* is larger right after *t* = 0 compared to later pump-probe delays. This feature is further echoed by the larger scan-to-scan variation at *t* = 3 μs (blue triangles) compared to *t* = 20 μs and 32 μs (red and green triangles) in Fig. 2e. This decrease in variation indicates that there is an underlying convergence of lattice motion at longer time delays compared to initial photoexcitation conditions, which suggests a correlation between grain conditions in the short-lived photoexcited regime compared to the long-time thermal relaxation, which we will characterize in the following section. Next, we turn to our second observation, in which the discontinuities in the time traces are often preceded or succeeded by a streak of similar values, as highlighted in dashed boxes in Fig. 2c, d. The presence of these streaks is unexpected if the stochastic lattice dynamics following each pump pulse are independent of each other. The streaks hint at some correlated photoinduced dynamics in LLTO. Both observations are not typically observed in simple thermal expansion and relaxation models without the presence of a phase transition or mobile charges modulating the lattice dynamics. We will quantify both features and relate each to the underlying physics of the photoinduced dynamics.

### Statistical analysis of lattice dynamics

We first examine the amplitude of the scan-to-scan *c* value variation that appears to decay as a function of delay time. Figure 3b confirms this observation, where the standard deviation (*c*_sd_) computed across scans exhibits a remarkably similar temporal evolution as that of the scan-averaged dynamics (*c*_avg_) in Fig. 3a. Both *c*_avg_ and *c*_sd_ can be well fitted to a phenomenological model (black curves in Fig. 3a, b), which captures essential features of a generic photoinduced response that approximately follows first-order kinetics during long-term recovery (with time constants *τ*_avg_ and *τ*_sd_)^42–44^,

1 $$f(t)={f}_{{\rm{equil}}}+\left[\frac{1}{2}\left(1+\,\rm{erf}\,\left(\frac{2\sqrt{\ln2}(t-{t}_{0})}{w}\right)\right)\cdot \left({I}_{\infty }+{I}_{0}{e}^{-(t-{t}_{0})/\tau }\right)\right],$$

where *f*(*t*) is the observable of interest that depends on the pump-probe delay time *t* obtained from the electronic delay signal, and *f*_equil_ is its equilibrium value prior to photoexcitation. *w* characterizes the initial system response time, *t*_0_ determines the pump pulse arrival time, *I*_0_ denotes the change right after photoexcitation, and *I*_∞_ denotes the value after the system partially relaxes, a process characterized by a time constant *τ*.

On the phenomenological level, the sudden increase of *c*_sd_(*t*) after *t* = 0 is indicative of the variation in the extent of the photoinduced lattice expansion, captured by *I*_0_ in Eq. (1). The physical origin of such variations in *I*_0_ can be the randomness of the strain environment^45^ following the mechanical relaxation of neighboring grains from the previous laser shot, the changing absorbed fluence due to varying light attenuation through a grainy medium where different grain orientations in the vicinity lead to different degrees of scattering, or a combination of such factors. However, a careful comparison between the dynamics of *c*_avg_(*t*) and *c*_sd_(*t*) in Fig. 3a, b indicates that *I*_0_ cannot be the only stochastic element that varies from shot to shot. Specifically, the relaxation time *τ*_avg_ is more than twice that of *τ*_sd_. This suggests a correlation between *I*_0_ and *τ* for each individual pump shot-induced response. Indeed, when we simulate a large number of lattice responses by drawing *I*_0_ randomly from a Gaussian distribution, we can reproduce the *τ*_avg_ > *τ*_sd_ relation (Fig. 3c, d) only if we impose a negative correlation between *I*_0_ and *τ*, where the exact functional form of the negative correlation is not critical (see Supplementary Note 6 for more details of the simulation). This insight, provided by reproducing the statistical signatures from the simulation, demonstrates a nonlinear force-displacement relationship, suggesting *c*-axis lattice stiffening in the thermally excited regime.

Next, we address the origin of the streaks in Fig. 2c, d, which suggest that variations in the lattice dynamics after each pump laser shot are not independent. To obtain a quantitative measure of the hidden dynamical correlation, we examine how the *c*-axis parameter within a scan at one particular delay time, *t*_*i*_, is correlated with its value at another delay time *t*_*j*_. Although we use delay times *t*_*i*,*j*_ as a convenient notation, the correlation we are interested in is defined between two lattice responses following two pump shots that arrive at different lab times; under our experimental condition, a delay difference ∣*t*_*i*_ − *t*_*j*_∣ of 1 μs corresponds to approximately 1000 pump shots elapsed (see measurement scheme in Fig. 2a). To the lowest order, we assume that a linear correlation can capture the relation, if any, between *c*(*t*_*i*_) and *c*(*t*_*j*_) across different pump shots. This assumption is expected to hold because a larger value of *c*(*t*_*i*_) at an early time delay indicates a larger initial lattice expansion, which in turn leads to a larger value of *c*(*t*_*j*_) during its microsecond relaxation period, where no coherent oscillatory dynamics were observed.

**Fig. 4:**
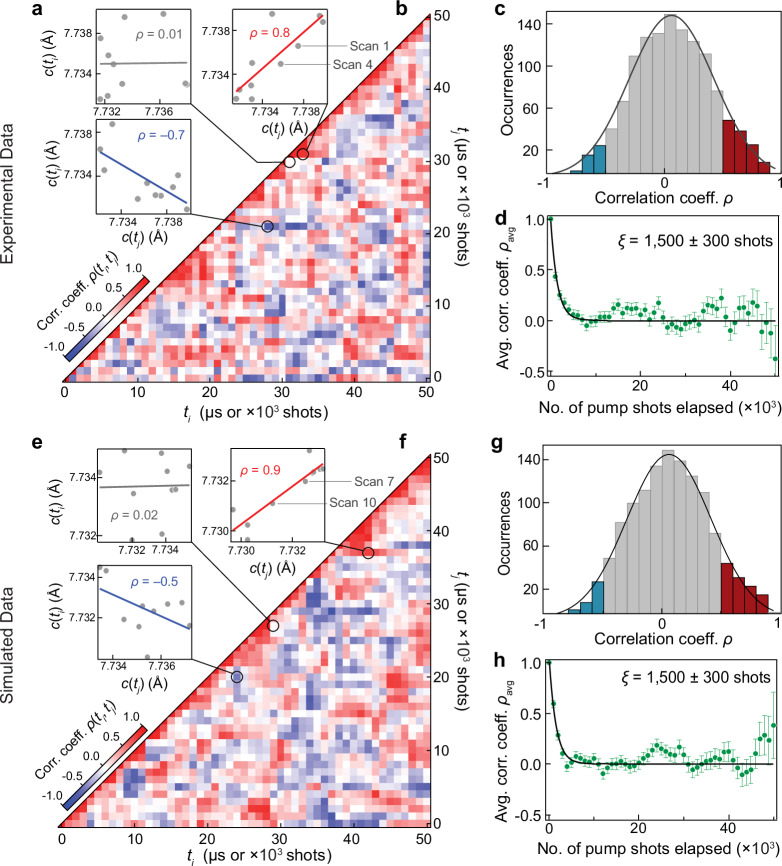
Hidden correlations between the shot-to-shot responses. **a**, **b** Pearson correlation *ρ*(*t*_*i*_, *t*_*j*_) of the measured lattice parameter *c* between two delay times *t*_*i*_, *t*_*j*_ ∈ [0, 50] μs, which are alternatively expressed as 10^3^ shots because 1 μs delay step in our sequential data acquisition scheme corresponds to 1000 pump laser shots incident on the sample. Each pixel in the two-time correlation matrix in (**b**) is derived from a scatter plot between *c*(*t*_*i*_) and *c*(*t*_*j*_). Three examples of positive, nearly zero, and negative Pearson correlation coefficients are shown in (**a**), where each gray dot represents the data point from one scan out of the 10 scans in Fig. 2c. The values in the two-time correlation matrix are nearly random except for a notable positive correlation along the 45° diagonal where *t*_*i*_ ≈ *t*_*j*_. **c** Histogram of all non-diagonal Pearson correlation coefficients in (**b**), where the solid curve is a Gaussian fit. The majority of the correlation coefficients are randomly distributed around zero, but there is a slight bias towards a positive value (highlighted by red vs. blue bars outside of ∣*ρ*∣ > 0.5) due to the large, positive correlations when *t*_*i*_ ≈ *t*_*j*_. **d** Averaged Pearson correlation coefficient *ρ*_avg_ as a function of ∣*t*_*i*_ − *t*_*j*_∣, the latter of which is expressed as the number of pump shots elapsed. Error bars denote the standard error of *ρ*_avg_ over 10 scans, and they become larger by a factor of $M_{| i-j| }^{-1/2}$ where *M*_∣*i*−*j*∣_ is the descending number of entries away from *t*_*i*_ = *t*_*j*_ in (**b**). Solid curve is a fit to an exponential decay, yielding a correlation length of *ξ* = 1, 500 ± 300 shots. **e**–**h** Simulated data corresponding to the measured results in panels (**a**–**d**), reproducing all key features. See the main text and Supplementary Note 6 for simulation details.

Based on the values of *c*(*t*_*i*_) and *c*(*t*_*j*_) in Fig. 2c, we computed the Pearson correlation coefficients^46 ^*ρ*(*t*_*i*_, *t*_*j*_) as a simple measure of their linear correlation: *ρ*(*t*_*i*_, *t*_*j*_) = cov[*c*(*t*_*i*_)*c*(*t*_*j*_)]/(*σ*[*c*(*t*_*i*_)]*σ*[*c*(*t*_*j*_)]), where the covariance (cov) and the standard deviation (*σ*) are computed across 10 scans. Similar to calculations commonly employed in the analysis of X-ray photon correlation spectroscopy (XPCS) data^47–49^, we represent correlations between *c*(*t*_*i*_) and *c*(*t*_*j*_) in a 2-D matrix, where each pixel is a unique mapping of *i* and *j*. This two-time correlation matrix is shown for its lower-half in Fig. 4b for all positive pump-probe time delays, where each pixel corresponds to a linear correlation coefficient derived from a scatter plot between *c*(*t*_*i*_) and *c*(*t*_*j*_) (Fig. 4a). Besides the *ρ*(*t*_*i*_, *t*_*j*=*i*_) ≡ 1 entries along the 45° diagonal, the two-time correlation matrix in Fig. 4b appears to be populated with mostly random values around *ρ* = 0. This randomness is reflected in the histogram of all non-diagonal entries in the correlation matrix (Fig. 4c), which can be fitted to a Gaussian distribution. We notice a small offset of the histogram towards positive *ρ*, which is best seen from the unbalanced tails of the histogram highlighted by red and blue bars. The excess positive values of *ρ* stem from the red features in the correlation matrix in the neighborhood of the 45° diagonal, indicating a high degree of correlation of the transient *c*-axis parameter if *t*_*i*_ ≈ *t*_*j*_; namely, pump-induced lattice trajectories are not independent if relatively few photoexcitation events have elapsed between the corresponding pump pulses. To quantify the correlation length *ξ* in terms of the elapsed pump shots during the measurement, we inspect how fast the Pearson correlation coefficient decays from 1 as one moves away from the 45° diagonal in the two-time correlation matrix. In practice, we compute an averaged correlation coefficient *ρ*_avg_(Δ*t*) by going through all *ρ*(*t*_*i*_, *t*_*j*_) that satisfies Δ*t* = ∣*t*_*i*_ − *t*_*j*_∣, shown in Fig. 4d. The resulting autocorrelation curve *ρ*_avg_ shows a clear exponentially decaying trend towards zero, yielding a correlation length of *ξ* = 1500 ± 300 shots, which is comparable to the number of pump shots received (*n* ≈ 1000 shots) at one specific delay during a scan in our data acquisition scheme. This statistical analysis hence gives a quantitative measure of how fast the dynamical correlation, which originates from the persistence behavior of the micro-grain, is lost as more photoexcitation events occur under repeated pump shots.

## Simulation and discussion

Despite the insight provided by our analysis above, we still require an understanding of the physical origin of the correlation in the stochastic lattice expansion and relaxation, as well as its connection to the underlying dynamics. To reveal the nature of the stochastic behavior, we simulated the measurements following the exact data acquisition scheme in the experiment (Fig. 2a). Using the fit function Eq. (1) from the averaged response in Fig. 3a, we employed the same phenomenological model to capture the system evolution after an individual photoexcitation event. In the simulation, stochastic responses are introduced by drawing the initial lattice response [*I*_0_ in Eq. (1)] randomly from a Gaussian distribution. However, if *I*_0_ is both random and independent after every pump shot, there is no dynamical correlation and the resulting *ρ*(*t*_*i*_, *t*_*j*_) matrix is truly randomly populated with no enhanced coefficients when *t*_*i*_ ≈ *t*_*j*_ (see Supplementary Note 6, Supplementary Fig. 7). To model the highly-correlated dynamics between neighboring shots, we introduce the following protocol: with probability *p*_0_ ≪ 1, *I*_0_ will be randomly selected for the next pump shot; otherwise, the photoinduced dynamics for the next shot will be identical to the dynamics following the current shot.

Despite the simplicity of our model, it recreated the key statistical properties observed in the experiment, such as the two-time correlation matrix and its histogram distribution (Fig. 4f, g), which look nearly indistinguishable from the experimental data (Fig. 4b, c). In individual time traces, important characteristics such as the discontinuities in between a stretch of continuous streaks are also clearly discernible (Supplementary Fig. 9). Similar to the experimental two-time correlation matrix in Fig. 4d, a high and positive correlation value is observed near the 45° diagonal where *t*_*i*_ ≈ *t*_*j*_, which decays to zero for large ∣*t*_*i*_ − *t*_*j*_∣ (Fig. 4h). In the simulation, the value of *p*_0_ was adjusted to yield the same correlation length of *ξ* = 1500 ± 300 shots, leading to *p*_0_ = (0.09 ± 0.02)%, where the error bar of *p*_0_ is computed to correspond to the lower and upper bounds of the correlation length *ξ*. Physically, *p*_0_ represents the probability for a micro-grain of LLTO to randomly change its nonequilibrium lattice expansion and relaxation trajectories for the next pump shot. The close agreement between the simulated and experimental data, particularly the exponential autocorrelation curve (Fig. 4d, h), suggests that the observed lattice trajectory switching can be described by a Markov chain, where future outcomes depend solely on the current state of the system rather than its accumulated history.

From an energetics perspective, the value of ${p}_{0}=\exp [-{E}_{0}/({\mathrm{k}}_{{\rm{B}}}T)]$ implies an energy barrier of *E*_0_ = 0.4 ± 0.1 eV, where k_B_ is the Boltzmann constant and *T* ≈ 620 K is the lattice temperature right after photoexcitation (see Supplementary Note 7 for an estimate of the photoinduced lattice temperature rise). We note that the value of *E*_0_ is close to both theoretical and experimental values of the energy barrier for lithium ion migration in LLTO (*E*_*b*_ ≈ 0.3–0.5 eV)^29,30,50^. Hence, a potential scenario is that lithium ion displacement after each pump pulse results in a slight variation in the metastable lattice structure.

To understand what can cause such a change in the metastable lattice, we note that ultraviolet light can induce a cascade of lattice dynamics, including Ti-O optical phonon modes and potentially the octahedral vibrational mode^51^. However, the excitation of these phonons in the harmonic limit, as in most other ultrafast experiments where both coherent and incoherent phonons have been observed, does not modify the temporally averaged lattice structure. Two exceptions that deviate from the harmonic limit include scenarios when (i) nonlinear phononic dynamics are induced via resonant driving at mid-IR or THz frequency of the pump light^52^ or (ii) a structural phase transition is induced^53^, neither of which is relevant for the present experiment in the absence of lithium ion hopping. Hence, while these modes can be excited, they cannot contribute to the observed stochastic dynamics of LLTO over microseconds. However, such modes are known to play a large role in lithium hopping through phonon-ion coupling^51,54^. While these modes are being excited, there is simultaneous interaction with the motion of lithium ions, which can result in a change in the temporally averaged lattice structure.

The photo-assisted lithium ion diffusion also offers an explanation for the negative correlation between the initial photoinduced lattice expansion [*I*_0_ in Eq. (1)] and the corresponding relaxation time *τ*. Such a relation typically shows up in optical pump-probe studies of semiconductors^55^ and superconductors^56^, where, in certain regimes, bimolecular recombination of electron-hole pairs or electron-electron pairs leads to a faster decay with a larger number of initially excited free carriers. In the case of LLTO, such mechanisms are not expected to apply, where relaxation over tens of microseconds is dictated by heat diffusion, strain relaxation, and macroscopic grain motion^21,57,58^. In the context of photoinduced structural change—especially through a nonequilibrium transition—a positive correlation between *I*_0_ and *τ* is typically observed^59–61^, contrary to our observation of LLTO. The positive *I*_0_-*τ* correlation in those other experiments^59–61^ is indicative of a soft mode as the system enters a flat potential energy landscape near the critical point. By contrast, in LLTO, lithium ion migration during the course of a photoinduced lattice evolution allows the lattice to enter a transient structure that is energetically more favorable than the one without any lithium ion movement. This process finds a local minimum by going away from the flat region in the potential energy surface, leading to a stiffer instead of softer lattice and hence accounting for the faster lattice relaxation.

Our results demonstrate a statistical method to track and characterize photoinduced dynamics that are not reproducible shot-to-shot over the course of a stroboscopic measurement. We expect this technique to be applicable to a variety of pump-probe experiments, provided that stochastic behavior can be conclusively assigned to the system, rather than experimental uncertainties. With fine-tuning of experimental parameters regarding the pulse repetition rate, data binning, and use of a highly stable pump-probe setup, correlations can be extracted from averaged data on a variety of timescales of interest. In the context of X-ray sciences, the nonequilibrium noise correlation spectroscopy demonstrated in this work presents a streamlined, complementary approach to photon correlation spectroscopy in the study of structural correlations at an ultrashort timescale while avoiding the technical complexity of implementing split X-ray pulses in a free-electron laser^62–64^. While XPCS can probe extremely small discontinuities over a large volume, our method offers a highly localized, low-damage probe that is necessary to access micro-scale physics. Our findings are relevant in the active pursuit of modeling and designing spatially heterogeneous or temporally fluctuating systems that underpin most materials of both fundamental and technological interest, such as unconventional superconductors^65,66^, self-assembled nanostructures^67^, and *in operando* catalysts^68^. In an intrinsically nonequilibrium context, such as biomolecular processes, our approach may help identify new structural dynamics and energy flow that are critical in understanding and orchestrating the reaction pathway^69^. This work provides a useful tool to characterize correlations in otherwise random dynamics that occur at the intrinsic timescale and length scale of electronic, ionic, and molecular motion.

## Methods

### Time-resolved X-ray micro-diffraction

Experiments were performed at the 7ID-C beamline at the Advanced Photon Source (APS) in Argonne National Laboratory, and the setup configurations were detailed in refs. ^70,71^. The geometry of the experiment is depicted in Fig. 1b and Supplementary Fig. 1. The samples were photoexcited above the bandgap of LLTO using a 3.55 eV (349 nm) femtosecond laser source, which was produced as the second harmonic of the output of an optical parametric amplifier (OPerA Solo, Coherent Inc.) using the output of an amplified Ti:sapphire laser (Legend, Coherent Inc.). The pump laser operates at 1 kHz repetition rate, which was locked to an integer division of the synchrotron repetition rate. The probing X-ray beam from the synchrotron had a pulse duration of 100 ps, operating at a 6.5 MHz repetition rate. The X-ray beam was then monochromatized to an energy of 8 keV (1.5498 Å) and focused by a pair of Kirkpatrick-Baez mirrors to a cross-sectional spot size of 12 μm × 15 μm (full-width at half maximum, FWHM), which was an order of magnitude smaller than the pump beam spot size at the sample position to ensure a near-uniform photoexcitation condition in the probed area. The powder pellet was mounted at the center of rotation of a six-circle diffractometer (Huber GmbH). The X-ray diffraction signals were collected by an area detector (Pilatus 100K, DECTRIS Ltd.), which was gated to selectively record the X-ray pulse that was paired with the excitation laser pulse, rendering the overall repetition rate of the experiment to 1 kHz. The time delay between the laser pump pulse and X-ray probe pulse was varied electronically, allowing us to access a timescale up to tens of microseconds, which is relevant for the slow relaxation process pertinent to heat dissipation through a grainy pellet^72^ as well as macroscopic grain motion as a result of laser-induced lattice parameter change. The diffraction pattern was stable and reproducible throughout the experiment. By scanning across the sample, regions corresponding to well-defined Bragg peaks were located and selected for the measurements.

### Sample preparation and characterization

Li_0.5_La_0.5_TiO_3_ was synthesized according to the procedures in ref. ^73^. A stoichiometric amount of La_2_O_3_, Li_2_CO_3_, and TiO_2_ was mixed in an agate mortar and pressed into pellets under 100 MPa of pressure. The pellets were placed on a bed of sacrificial powder and calcined at 800 ^°^C for 4 h, then at 1200 °C for 12 h at a ramp rate of 1 °C/min. After the crystal structure of LLTO was confirmed with X-ray diffraction, the resulting powder was pressed into a pellet with a diameter of 6 mm and a thickness of 0.5–1 mm under 620 MPa of pressure. The pellet was subsequently annealed at 1100 °C for 6 h at a ramp rate of 2 °C/min over a bed of its mother powder. To characterize the grain morphology, scanning electron microscopy (SEM) was performed using the SE2 detector of a ZEISS 1550VP field emission SEM with an acceleration voltage of 10 kV at 10k× magnification. As shown in Fig. 1c, the grain size of LLTO is comparable to the X-ray beam spot size for the time-resolved X-ray micro-diffraction measurements.

## Supplementary information

Supplementary Information

Transparent Peer Review file

## Data Availability

All of the data and calculations supporting the conclusions are available within the article and the Supplementary Information. The raw and processed data generated in this study, as well as the Igor Pro 8 code for processing the data and full simulation, have been deposited in the Dryad database [10.5061/dryad.dfn2z35gt].

## References

[1] Moffat, K. Ultrafast time-resolved crystallography. *Nat. Struct. Mol. Biol.* **5**, 641–643 (1998). 10.1038/1333 9699613

[2] Torre, A. et al. Colloquium: nonthermal pathways to ultrafast control in quantum materials. *Rev. Mod. Phys.* **93**, 041002 (2021).

[3] Zong, A., Nebgen, B. R., Lin, S.-C., Spies, J. A. & Zuerch, M. Emerging ultrafast techniques for studying quantum materials. *Nat. Rev. Mater.* **8**, 224–240 (2023).

[4] Lépine, F., Ivanov, M. Y. & Vrakking, M. J. J. Attosecond molecular dynamics: fact or fiction? *Nat. Photon.* **8**, 195–204 (2014).

[5] Mo, M. Z. et al. Single-shot mega-electronvolt ultrafast electron diffraction for structure dynamic studies of warm dense matter. *Rev. Sci. Instrum.* **87**, 11–810 (2016). 10.1063/1.4960070 27910490

[6] Liang, J. & Wang, L. V. Single-shot ultrafast optical imaging. *Optica* **5**, 1113 (2018). 30820445 10.1364/OPTICA.5.001113 PMC6388706

[7] Helk, T., Zürch, M. & Spielmann, C. Perspective: towards single-shot time-resolved microscopy using short-wavelength table-top light sources. *Struct. Dyn.* **6**, 010902 (2019). 30868083 10.1063/1.5082686 PMC6404932

[8] Wakeham, G. P. & Nelson, K. A. Dual-echelon single-shot femtosecond spectroscopy. *Opt. Lett.* **25**, 505 (2000). 18064094 10.1364/ol.25.000505

[9] Shin, T., Wolfson, J. W., Teitelbaum, S. W., Kandyla, M. & Nelson, K. A. Dual echelon femtosecond single-shot spectroscopy. *Rev. Sci. Instrum.* **85**, 083115 (2014). 25173254 10.1063/1.4893641

[10] Noe, G. T. et al. Single-shot terahertz time-domain spectroscopy in pulsed high magnetic fields. *Opt. Express* **24**, 30328 (2016). 28059309 10.1364/OE.24.030328

[11] Yu, L. et al. A comprehensive review of fluorescence correlation spectroscopy. *Front. Phys.* **9**, 644450 (2021).

[12] Wang, Q. et al. A comprehensive overview of diffuse correlation spectroscopy: theoretical framework, recent advances in hardware, analysis, and applications. *NeuroImage* **298**, 120793 (2024). 39153520 10.1016/j.neuroimage.2024.120793

[13] Yin, H. et al. Theory of x-ray photon correlation spectroscopy for multiscale flows. *Phys. Rev. Res.* **7**, 023202 (2025).

[14] Wall, S. et al. Ultrafast disordering of vanadium dimers in photoexcited VO_2_. *Science* **362**, 572–576 (2018). 30385575 10.1126/science.aau3873

[15] Zong, A. et al. Role of equilibrium fluctuations in light-induced order. *Phys. Rev. Lett.* **127**, 227401 (2021). 34889631 10.1103/PhysRevLett.127.227401

[16] Perez-Salinas, D., Johnson, A. S., Prabhakaran, D. & Wall, S. Multi-mode excitation drives disorder during the ultrafast melting of a C4-symmetry-broken phase. *Nat. Commun.* **13**, 238 (2022). 35017507 10.1038/s41467-021-27819-y PMC8752725

[17] Disa, A. S. et al. Photo-induced high-temperature ferromagnetism in YTiO_3_. *Nature* **617**, 73–78 (2023). 37138109 10.1038/s41586-023-05853-8 PMC10156606

[18] Mcleod, A. S. et al. Multi-messenger nanoprobes of hidden magnetism in a strained manganite. *Nat. Mater.* **19**, 397–404 (2020). 31844275 10.1038/s41563-019-0533-y

[19] Zhang, J. et al. Cooperative photoinduced metastable phase control in strained manganite films. *Nat. Mater.* **15**, 956–960 (2016). 27400387 10.1038/nmat4695

[20] Liu, Q. et al. Photoinduced multistage phase transitions in Ta_2_NiSe_5_. *Nat. Commun.* **12**, 2050 (2021). 33824351 10.1038/s41467-021-22345-3 PMC8024274

[21] Stoica, V. A. et al. Non-equilibrium pathways to emergent polar supertextures. *Nat. Mater.* **23**, 1394–1401 (2024). 39317816 10.1038/s41563-024-01981-2

[22] Ilyas, B. et al. Terahertz field-induced metastable magnetization near criticality in FePS_3_. *Nature* **636**, 609–614 (2024). 39695209 10.1038/s41586-024-08226-x

[23] Padma, H. et al. Symmetry-protected electronic metastability in an optically driven cuprate ladder. *Nat. Mater.* **24**, 1584–1591 (2025). 40461831 10.1038/s41563-025-02254-2

[24] Stramare, S., Thangadurai, V. & Weppner, W. Lithium lanthanum titanates: a review. *Chem. Mater.* **15**, 3974–3990 (2003).

[25] Belous, A. et al. Peculiarities of Li_0.5_La_0.5_TiO_3_ formation during the synthesis by solid-state reaction or precipitation from solutions. *Chem. Mater.* **16**, 407–417 (2004).

[26] Nakayama, M., Usui, T., Uchimoto, Y., Wakihara, M. & Yamamoto, M. Changes in electronic structure upon lithium insertion into the *A*-site deficient perovskite type oxides (Li, La)TiO_3_. *J. Phys. Chem. B* **109**, 4135–4143 (2005). 16851474 10.1021/jp046062j

[27] Qian, D. et al. Lithium lanthanum titanium oxides: a fast ionic conductive coating for lithium-ion battery cathodes. *Chem. Mater.* **24**, 2744–2751 (2012).

[28] Okumura, T. et al. Effect of average and local structures on lithium ion conductivity in La_2/3−*x*_Li_3*x*_TiO_3_. *J. Mater. Chem.* **21**, 10195–10205 (2011).

[29] Zhang, L. et al. Lithium lanthanum titanate perovskite as an anode for lithium-ion batteries. *Nat. Commun.* **11**, 3490 (2020). 32661230 10.1038/s41467-020-17233-1 PMC7359355

[30] Woodahl, C. et al. Probing lithium mobility at a solid electrolyte surface. *Nat. Mater.* **22**, 848–852 (2023). 37106132 10.1038/s41563-023-01535-y PMC10313518

[31] Cotret, L. P. et al. Time- and momentum-resolved phonon population dynamics with ultrafast electron diffuse scattering. *Phys. Rev. B* **100**, 214115 (2019).

[32] Cheng, Y. et al. Ultrafast formation of topological defects in a two-dimensional charge density wave. *Nat. Phys.* **20**, 54–60 (2024).

[33] Sokolowski-Tinten, K., Bialkowski, J. & Linde, D. Ultrafast laser-induced order-disorder transitions in semiconductors. *Phys. Rev. B* **51**, 14186–14198 (1995). 10.1103/physrevb.51.14186 9978346

[34] Shugaev, M. V. et al. Fundamentals of ultrafast laser-material interaction. *MRS Bull.* **41**, 960–968 (2016).

[35] Yashima, M., Itoh, M., Inaguma, Y. & Morii, Y. Crystal structure and diffusion path in the fast lithium-ion conductor La_0.62_Li_0.16_TiO_3_. *J. Am. Chem. Soc.* **127**, 3491–3495 (2005). 15755169 10.1021/ja0449224

[36] Kim, T. et al. Mapping the pathways of photo-induced ion migration in organic-inorganic hybrid halide perovskites. *Nat. Commun.* **14**, 1846 (2023). 37012242 10.1038/s41467-023-37486-w PMC10070404

[37] Wakamura, K. Effects of electronic band on activation energy and of effective charge on lattice distortion in superionic conductors. *J. Phys. Chem. Solids* **59**, 591–598 (1998).

[38] Rahaman, M.N. Ceramic Processing and Sintering, 2nd edn. (CRC Press, Boca Raton, FL, 2017).

[39] Matzen, S. et al. Tuning Ultrafast Photoinduced Strain in Ferroelectric-Based Devices. *Adv. Electron. Mater.* **5**, 1800709 (2019).

[40] Sharif, M., Ma, X. & Ghadiri, E. Visualization and deconvolution of carrier kinetics within grains of Cu_2_ZnSnS_4−*x*_Se_*x*_ using ultrafast diffuse reflectance microscopy. *J. Mater. Chem. C.* **12**, 12254–12265 (2024).

[41] Tasca, K. R. et al. Time-resolved X-ray powder diffraction study of photoinduced phase transitions in Ti_3_O_5_ nanoparticles. *Chem. Phys. Chem.* **18**, 1385–1392 (2017). 28220594 10.1002/cphc.201601337

[42] Moore, R. G. et al. Ultrafast resonant soft X-ray diffraction dynamics of the charge density wave in TbTe_3_. *Phys. Rev. B* **93**, 024304 (2016).

[43] Zong, A. Emergent states in photoinduced charge-density-wave transitions. (Springer, Cham, 2021).

[44] Demsar, J., Dekorsy, T.: 8. In: Prasankumar, R.P., Taylor, A.J. (eds.) Carrier dynamics in bulk semiconductors and metals after ultrashort pulse excitation, 291–328. (CRC Press, Boca Raton, 2012).

[45] Rubio-Marcos, F. et al. Photocontrolled strain in polycrystalline ferroelectrics via domain engineering strategy. *ACS Appl. Mater. Interfaces* **13**, 20858–20864 (2021). 33881295 10.1021/acsami.1c03162 PMC8480775

[46] Boslaugh, S. Statistics in a nutshell, 2nd edn. (O’Reilly Media, Sebastopol, CA, 2012)

[47] Ragulskaya, A., Starostin, V., Zhang, F., Gutt, C. & Schreiber, F. On the analysis of two-time correlation functions: equilibrium versus non-equilibrium systems. *Appl. Crystallogr.* **57**, 1098–1106 (2024). 10.1107/S1600576724004618 PMC11299609 39108815

[48] Jain, A. et al. Three-step colloidal gelation revealed by time-resolved x-ray photon correlation spectroscopy. *J. Chem. Phys.* **157**, 184901 (2022). 36379773 10.1063/5.0123118

[49] Chushkin, Y. et al. Probing cage relaxation in concentrated protein solutions by X-ray photon correlation spectroscopy. *Phys. Rev. Lett.* **129**, 238001 (2022). 36563210 10.1103/PhysRevLett.129.238001

[50] Geng, H. X., Mei, A., Dong, C., Lin, Y. H. & Nan, C. W. Investigation of structure and electrical properties of Li_0.5_La_0.5_TiO_3_ ceramics via microwave sintering. *J. Alloy. Compd.* **481**, 555–558 (2009).

[51] Pham, K. H. et al. Correlated terahertz phonon-ion interactions control ion conduction in a solid electrolyte. *Mater. Horiz.* **13**, 3355–3375 (2026). 41603889 10.1039/d5mh01990g

[52] Först, M. et al. Nonlinear phononics as an ultrafast route to lattice control. *Nat. Phys.* **7**, 854–856 (2011).

[53] Johnson, A. S. et al. Ultrafast X-ray imaging of the light-induced phase transition in VO_2_. *Nat. Phys.* **19**, 215–220 (2023).

[54] Pham, K. H. et al. The dynamical role of optical phonons and sublattice screening in a solid-state ion conductor. *J. Am. Chem. Soc.* **147**, 26456–26467 (2025). 40674462 10.1021/jacs.5c06064

[55] Shkrob, I. A. & Crowell, R. A. Ultrafast charge recombination in undoped amorphous hydrogenated silicon. *Phys. Rev. B* **57**, 12207–12218 (1998).

[56] Gedik, N. et al. Single-quasiparticle stability and quasiparticle-pair decay in YBa_2_Cu_3_O_6.5_. *Phys. Rev. B* **70**, 014504 (2004).

[57] Cherukara, M. J. et al. Ultrafast three-dimensional X-ray imaging of deformation modes in ZnO nanocrystals. *Nano Lett.* **17**, 1102–1108 (2017). 28026962 10.1021/acs.nanolett.6b04652

[58] Chen, G. Nanoscale Energy Transport and Conversion: A Parallel Treatment of Electrons, Molecules, Phonons, and Photons. Oxford University Press: Oxford, 2005.

[59] Zong, A. et al. Evidence for topological defects in a photoinduced phase transition. *Nat. Phys.* **15**, 27–31 (2019).

[60] Huber, T. et al. Coherent structural dynamics of a prototypical charge-density-wave-to-metal transition. *Phys. Rev. Lett.* **113**, 026401 (2014). 25062214 10.1103/PhysRevLett.113.026401

[61] Beaud, P. et al. A time-dependent order parameter for ultrafast photoinduced phase transitions. *Nat. Mater.* **13**, 923–927 (2014). 25087068 10.1038/nmat4046

[62] Osaka, T. et al. Wavelength-tunable split-and-delay optical system for hard X-ray free-electron lasers. *Opt. Express* **24**, 9187–9201 (2016). 27137535 10.1364/OE.24.009187

[63] Roseker, W. et al. Towards ultrafast dynamics with split-pulse X-ray photon correlation spectroscopy at free electron laser sources. *Nat. Commun.* **9**, 1704 (2018). 29703980 10.1038/s41467-018-04178-9 PMC5923200

[64] Zhu, D. et al. Development of a hard X-ray split-delay system at the Linac Coherent Light Source. In *Advances in X-ray Free-Electron Lasers Instrumentation.* IV, 103–110. (SPIE, Bellingham, WA, 2017)

[65] Torchinsky, D. H., Mahmood, F., Bollinger, A. T., Božović, I. & Gedik, N. Fluctuating charge-density waves in a cuprate superconductor. *Nat. Mater.* **12**, 387–391 (2013). 23435216 10.1038/nmat3571

[66] Arpaia, R. et al. Dynamical charge density fluctuations pervading the phase diagram of a Cu-based high-*T*_*c*_ superconductor. *Science* **365**, 906–910 (2019). 31467219 10.1126/science.aav1315

[67] Ekiz, M. S., Cinar, G., Khalily, M. A. & Guler, M. O. Self-assembled peptide nanostructures for functional materials. *Nanotechnology* **27**, 402002 (2016). 27578525 10.1088/0957-4484/27/40/402002

[68] Johánek, V. et al. Fluctuations and bistabilities on catalyst nanoparticles. *Science* **304**, 1639–1644 (2004). 15131265 10.1126/science.1097513

[69] Bhowmick, A. et al. Structural evidence for intermediates during O_2_ formation in photosystem II. *Nature* **617**, 629–636 (2023). 37138085 10.1038/s41586-023-06038-z PMC10191843

[70] Walko, D. A. et al. Developments in time-resolved X-ray research at APS beamline 7ID. *AIP Conf. Proc.* **1741**, 030048 (2016).

[71] Zhou, F. et al. Dynamical criticality of spin-shear coupling in van der Waals antiferromagnets. *Nat. Commun.* **13**, 6598 (2022). 36329063 10.1038/s41467-022-34376-5 PMC9633802

[72] Han, Q. et al. Additive engineering for high-performance room-temperature-processed perovskite absorbers with micron-size grains and microsecond-range carrier lifetimes. *Energy Environ. Sci.* **10**, 2365–2371 (2017).

[73] Inaguma, Y., Chen, L., Itoh, M. & Nakamura, T. Candidate compounds with perovskite structure for high lithium ionic conductivity. *Solid State Ion.* **70-71**, 196–202 (1994).

[74] Momma, K. & Izumi, F. VESTA 3 for three-dimensional visualization of crystal, volumetric and morphology data. *J. Appl. Crystallogr.* **44**, 1272–1276 (2011).

