# Supplementary material for: Photoinduced correlations in stochastic dynamics of a solid-state ionic conductor
Source: Nat Commun. 2026 May 15;17:6552. doi: 10.1038/s41467-026-72663-7 (PMC13381525; doi:10.1038/s41467-026-72663-7)
Supplement: Supplementary file 1 — Supplementary Information [file 41467_2026_72663_MOESM1_ESM.pdf]

Supplementary Information for "Photoinduced correlations in stochastic dynamics of a solid-state ionic conductor"

Supplementary Note 1. Determination of lattice parameters

We presented the photoinduced changes to the c lattice parameter in Fig. 2c in the main text, and here we explain how c was calculated based on the angle of the diffracted X-rays. The raw diffraction data consists of a series of two-dimensional images of the diffraction peak (Fig. 1d), where the position and intensity are recorded for each time delay. The diffracted peak is first fit with a 2D Gaussian function to retrieve the peak position with sub-pixel accuracy. This peak position on the pixel-array camera is then converted into lab coordinates to retrieve the position and angle of the peak relative to the sample. This coordinate conversion is done by applying a series of rotation matrices to convert the peak position in the camera frame axes (x', y', z') to the lab frame axes (x, y, z) (see Suppl. Fig. 1), as detailed below.

Let us denote the position of the peak in the camera frame as $(\Delta x', \Delta y', z'_0)$. Here, Δ implies that we calculate the peak position (in mm) relative to a reference central pixel

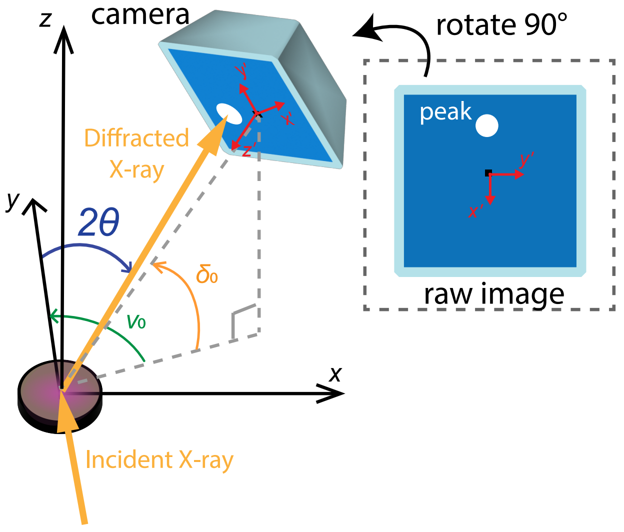

Suppl. Fig. 1. Schematic of the diffraction geometry, showing the lab frame coordinates (x, y, z) and the camera frame coordinates (x', y', z'). Incoming X-rays propagate along $+y$. The small black square on the camera screen is the reference central pixel, denoting the direct X-ray beam position when $\delta_0 = \nu_0 = 0$, where δ_0 and ν_0 are the rotations of the detector about the horizontal and vertical axes, respectively.

where $z_0' = -401$ mm is the distance between the sample and the reference central pixel, and δ_0 and ν_0 are the angles defined with respect to this reference central pixel as well. The negative sign in z_0' is added because it is computationally more convenient to define the origin of the camera coordinates at the sample position instead of in the plane of the camera. Under this definition, Δx and Δy are given by

$$\Delta x' = C_0(y_p' - y_0'), \qquad \Delta y' = C_0(x_p' - x_0'), \tag{S1}$$

where $C_0 = 0.172$ mm/pixel is the physical size of the pixel, $(x_0', y_0') = (327, 95)$ (in pixels) are the central pixel coordinates, and (x_p', y_p') (in pixels) are the diffraction peak coordinates. The x and y labels are swapped because the camera is rotated 90° relative to the saved raw images (see Suppl. Fig. 1).

Under these definitions, we can now apply rotation matrices to convert the lab frame to the camera frame and vice versa. This conversion was done by first rotating the lab frame axes (x, y, z) by angle $-\nu_0$ about the z axis and then by $(270° - \delta_0)$ about the x axis to overlap the coordinate axes seen on the camera. The two rotation matrices are

$$R_x(\phi) = \begin{pmatrix} 1 & 0 & 0 \\ 0 & \cos\phi & -\sin\phi \\ 0 & \sin\phi & \cos\phi \end{pmatrix}, \qquad R_z(\phi) = \begin{pmatrix} \cos\phi & \sin\phi & 0 \\ -\sin\phi & \cos\phi & 0 \\ 0 & 0 & 1 \end{pmatrix}, \tag{S2}$$

and the camera coordinates of the diffraction peak are related to its lab-frame position (x_0, y_0, z_0) as

$$(x_0, y_0, z_0)^T = R_z^{-1}(-\nu_0)\, R_x^{-1}(270° - \delta_0)\, (\Delta x', \Delta y', -z_0')^T. \tag{S3}$$

After we solve for (x_0, y_0, z_0), the diffraction angle 2θ is given by

$$2\theta = \arccos\left(y_0 / \sqrt{x_0^2 + y_0^2 + z_0^2} \right). \tag{S4}$$

For a two-dimensional detector screen, to the lowest order, 2θ informs us about the c lattice parameter if one measures a $(00L)$ diffraction peak. However, after photoexcitation, the crystalline micro-grain can have a slight rotation that moves the reciprocal lattice site

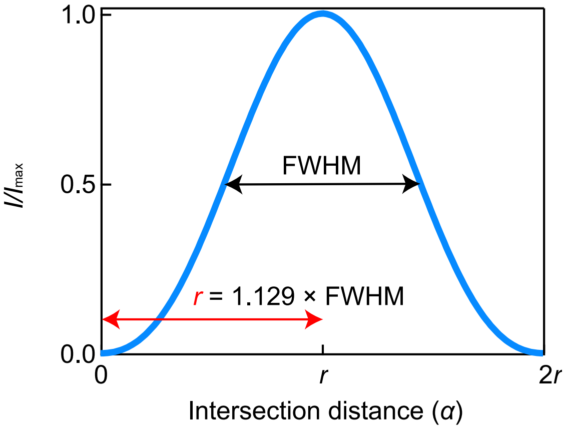

Suppl. Fig. 2. Profile of integrated intensity of a diffraction peak as a function of where the reciprocal lattice site (RLS) intersects the Ewald's sphere (see Suppl. Fig. 3). The FWHM is related to the characteristic size l_0 of the grain, from which a characteristic radius of the RLS $r \sim 1/l_0$ can be estimated.

(RLS) away from the Ewald's sphere and hence leads to a different integrated peak intensity, necessitating a slight revision of the 2θ value. To account for this effect, consider an Ewald's sphere with a radius $R = 2\pi/\lambda$, where $\lambda = 1.5498$ Å is the wavelength of the incoming X-ray beam. The characteristic radius of the RLS is given by $r \sim 2\pi/l_0$, where l_0 is the characteristic size of the grain under investigation, and this finite-size effect leads to a diffraction peak intensity profile following a sinc-squared function as depicted in Suppl. Fig. 2.

To illustrate the impact of this small deviation from the perfect Bragg condition on our derivation of the c lattice parameter based on a $(00L)$ peak, as illustrated in Suppl. Fig. 3, we define α as the reciprocal space distance between the characteristic perimeter of the RLS and its intersection plane with the Ewald's sphere (see Suppl. Fig. 3 inset). The observed integrated intensity I can then be related to the maximum possible intensity $I_{\max}$ under the perfect Bragg condition by

$$I = \frac{\sin^2[\pi(\alpha - r)/r]}{[\pi(\alpha - r)/r]^2} I_{\max}. \tag{S5}$$

From our experiments, $I_{\max} = 1,400$ counts for the (004) peak shown in Fig. 2c in the main text. r is defined from the peak center to the first zero of the sinc-squared function, and it is related to the full-width at half maximum of the diffraction peak by a numerical factor (see Suppl. Fig. 2). These values of r and $I_{\max}$ allow us to solve for α for each measured integrated intensity I of a $(00L)$ peak, and the geometry in Suppl. Fig. 3 yields the expression for the

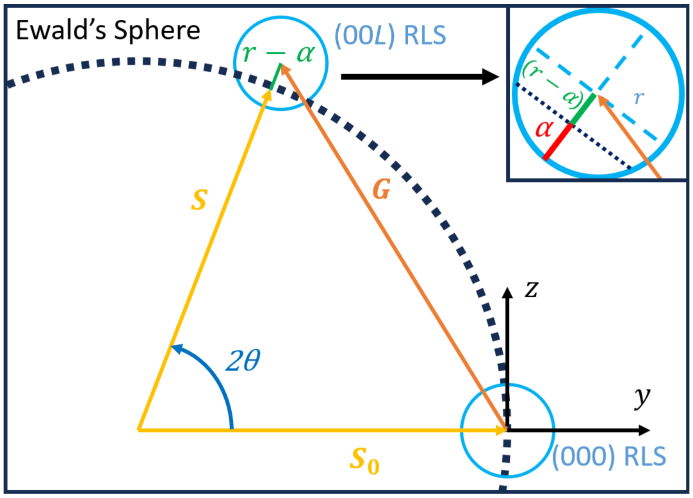

Suppl. Fig. 3. Schematic of a deviation from the ideal Bragg condition. The two reciprocal lattice sites (RLSs) are denoted by the blue circles with a characteristic radius r separated by reciprocal lattice vector $\mathbf{G}$, where $|\mathbf{G}| = Lc^*$ for a $(00L)$ peak and $c^* \equiv 2\pi/c$. Diffraction peaks are visible when the RLS intersects with the Ewald's sphere defined by the incoming and outgoing X-ray beams $\mathbf{S}_0$ and $\mathbf{S}$, respectively. α is related to the deviation of the perfect Bragg condition, which occurs at $\alpha = r$.

lattice parameter $c \equiv 2\pi/c^*$, where

$$(Lc^*)^2 = R^2 + (R + r - \alpha)^2 - 2R(R + r - \alpha)\cos 2\theta. \tag{S6}$$

Here, $R \equiv |\mathbf{S}_0|$, and $r = 3.55 \times 10^{-3}$ Å^{-1} from the peak profile on the camera.

The computed c value in Fig. 2c is approximately 0.4% smaller than the literature values reported in refs.[1,2], and the discrepancy may arise from the strained condition associated with the particular micro-grain under investigation in a pressed powder pellet.

Supplementary Note 2. Long-time drift correction

In a number of data sets, we observed a small but notable long-time drift of the lattice parameters across different scans, which may be attributed to environmental instability or long term strain relaxation of the micro-grain. Since we are concerned with the stochastic dynamics on the timescale that is much shorter than the time taken to complete ten scans (about ten minutes), this drift should be corrected before computing any statistical correlations. Otherwise, the correlation matrix in Fig. 4b will be dominated by an artificially positive correlation arising from the scan-to-scan long-term drift. We corrected such drift

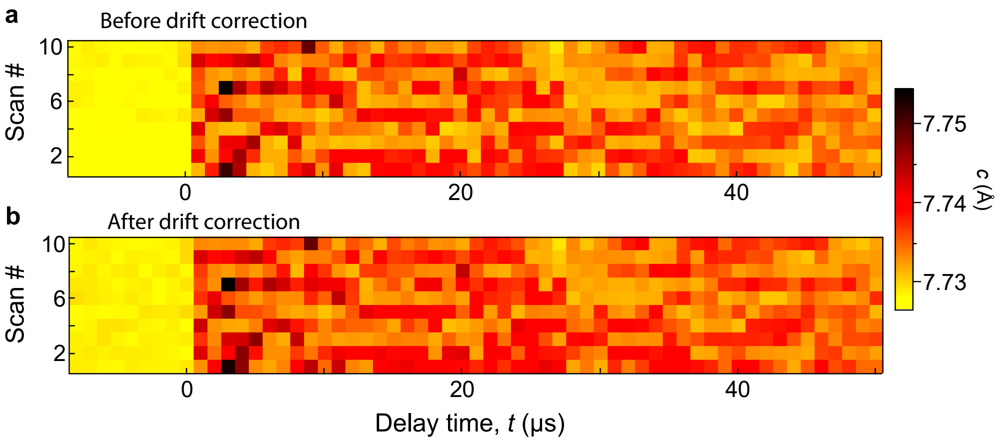

Suppl. Fig. 4. Measured c lattice parameter before (**a**) and after (**b**) drift correction, where the correction adds an offset to c at all time points in a particular scan such that the averaged c values before photoexcitation are the same across all scans. Colors are reversed from Fig. 2c to highlight the slight differences before time zero.

by offsetting all values in a scan such that the pre-timezero averages are the same across all scans. Figure 4 compares the dataset shown in Fig. 2c before and after the drift correction, demonstrating that the correction is indeed small ($\approx 1 \times 10^{-3}$ Å) compared to the stochastic response. Hence, the procedure has no major impact on our conclusions in the main text.

Supplementary Note 3. Noise analysis on a comparative study of lead zirconate titanate

As mentioned in the main text, a time-resolved X-ray diffraction experiment on $Pb(Zr_{0.2}Ti_{0.8})O_3$ or PZT, was conducted at the same beamline (7ID-C at Advanced Photon Source) as our experiment under similar conditions[3]. In PZT, we would not expect to see a stochastic response because there are no large mobile charge carriers in PZT which could cause a permanent lattice distortion between pulse events. In LLTO, we expect lithium migration to be a leading factor in the stochastic nature of the sample response to light. The PZT study was performed under nearly identical conditions, using 320 nm pump at an absorbed fluence of 6.22 mJ/cm^2, and X-ray wavelength of 1.241 Å. Similar to LLTO, PZT shows pump-induced expansion and contraction of the lattice. However, the noise before and after time zero is nearly identical, as indicated in Suppl. Fig. 5a below. The residual data from the fit was compared to the fluctuations of LLTO, where the noise increases significantly after time zero as seen in Fig. 3b. This indicates that the observed phenomenon

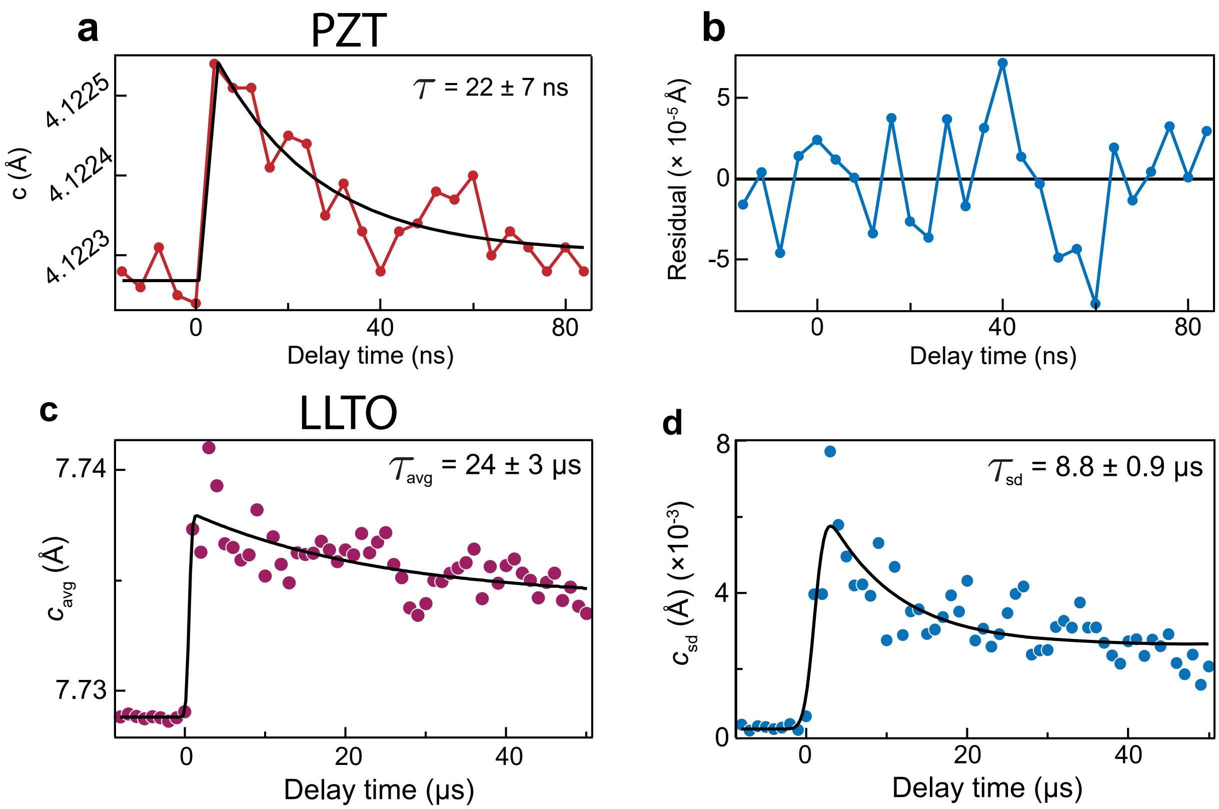

Suppl. Fig. 5. **a** The c lattice parameter of PZT as a function of pump-probe time delay with the black curve indicating the lattice relaxation trend. Data was replotted from Matzen et al.[3] with permissions. Fluctuations in this data are characterized by analyzing the differences between c and the value of the fit, plotted in panel **b**. The standard deviation before and after time zero are calculated to be $(2.5 \pm 1.1) \times 10^{-5}$ Å and $(3.5 \pm 0.8) \times 10^{-5}$ Å, respectively, indicating that there is negligible difference in stochastic sample response in PZT due to a pump pulse. Importantly, the residual data in panel **b** shows no clear signature of time zero (i.e., pump pulse arrival), in stark contrast to the noise structure in the LLTO data (see Fig. 2d of the main text). **c** and **d** The average and standard deviation of the LLTO c lattice parameter as a function of time for comparison.

in LLTO is not due to the beamline conditions but rather intrinsic to the sample itself. This comparative study further strengthens the core finding of our measurements on LLTO about the unusual stochastic response after UV photoexcitation.

Supplementary Note 4. Comparison of instrumental uncertainty and sample-intrinsic fluctuations

In the main text, we compared the noise level in the measured c lattice parameter before and after photoexcitation (Fig. 2c,d). Here, the fluctuations in c before time zero reflect the

instrumental uncertainty during the data collection process, assuming the micro-grain has fully relaxed during the 1 ms interval between successive pump pulses. On the other hand, the variation of the c value after time zero also encodes information about the intrinsically stochastic sample response. For a quantitative comparison of the noise levels, we first fit the time evolution of c using the phenomenological model described by Eq. (1), which captures well the averaged response (Fig. 3a). The residual data points were then grouped into points before and after time zero, and their respective standard deviations were calculated and compared.

To eliminate pump fluctuations as the source of noise after time zero, we compared the relative standard deviation of the fluctuations in LLTO with our pump laser. Quantitatively, we calculated the coefficient of variation (CV), defined as the standard deviation of the trace divided by the average (CV = σ/μ). In the time trace of the c parameter of LLTO, CV was calculated at each time point across all scans presented in Suppl. Fig. 6a. It is worth mentioning that since we are considering the photoinduced change of the c lattice constant, we first subtracted the static pre-time zero factor of 7.728 Å. Next, we extracted CV from a pump laser power trace, where the power as a function of laser shots was binned to match the acquisition of the LLTO c parameter. Figure 6b tabulates the coefficient of variation of both traces as a percentage. It is clear from this table that the fluctuations in LLTO are much higher compared to typical pump laser fluctuations, which is a good indicator that pump laser cannot cause the observed fluctuations in the photoinduced dynamics.

One remaining possibility is that any small fluctuation of the pump laser energy will lead to a large change in the lattice response. To explore this possibility, we present a power-dependent change in the steady-state lattice constant (Suppl. Fig. 6c) taken at the time of the experiment which shows a linear trend of the c lattice displacement as a function of pump power. This displacement is the difference from its reference position when there is no pump illumination. The linear trend suggests that there are negligible non-linear processes in LLTO that could amplify the noise of the pump laser and lead to the observed c parameter trajectory switching. This analysis hence suggests that fluctuations of the pump laser are not the dominant processes that lead to the stochastic lattice response observed in LLTO.

a

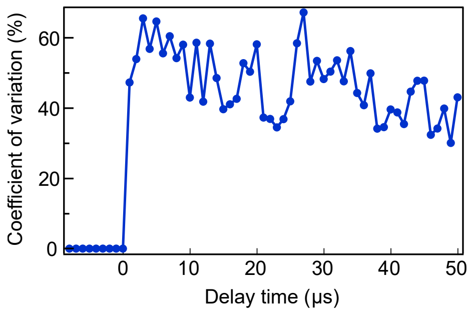

b

Trace	CV (%)
LLTO	≈ 40-70
349 nm	1.6

c

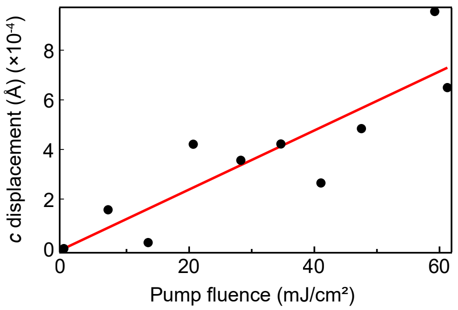

Suppl. Fig. 6. **a** Time evolution of the coefficient of variation (CV) of fluctuations of the c lattice parameter as a function of time. This was calculated by dividing the standard deviation (Fig. 3b) by the change in the average trace (Fig. 3a). **b** Coefficient of variation observed in the power fluctuations of the pump laser compared to fluctuations of the c parameter of LLTO, which displays more than an order of magnitude difference. **c** Fluence dependent change in the c parameter relative to no pump illumination. The plot shows a linear dependence in power fluctuations suggesting that fluctuations in pump power are not amplified by the sample response.

Supplementary Note 5. Energy absorbed by a single LLTO grain from pump laser

This section will approximate the energy absorbed per volume of a single grain to ensure there are no unforeseen phenomena such as lattice melting that could result from an excessive absorbed energy density. First, we calculate the penetration depth of the 349 nm light using Eq. (S7)[4].

$$\delta = \frac{c}{2\kappa\omega}, \tag{S7}$$

where c is the speed of light, κ is the imaginary part of the complex refractive index[5], and ω is the angular frequency of the pump laser light[5]. From this we find that $\delta \approx 30$ nm. This is small compared to the size of the illuminated grain (≈ 1 μm). On the other hand, the attenuation length of the probing X-ray is longer than the size of a typical grain. Hence, at our timescale of interest, the measured diffraction is a grain-averaged observation assuming that the energy deposited by the pump laser is averaged over the entire grain. Next, we can approximate the absorbed energy per unit volume of a single grain by Eq. (S8)[4].

$$\Upsilon = \frac{F_{\text{in}}}{d}(1 - R), \tag{S8}$$

where Υ is in units of mJ/cm^3, F_{in} is the incident fluence (67 mJ/cm^2), R is the normal-incidence reflectivity at 349 nm (calculated using the wavelength-dependent complex refractive index of LLTO[5]), and d is the typical grain size ($\approx$ 1 μm). Note that Eq. (S8) only gives an upper bound approximation because it assumes a flat surface of the grain, but realistically the grain has a curved and rugged surface which will result in less absorption. From this equation, we find the energy absorbed density by a single grain is upper bounded by 4.7×10^{-7} mJ/μm^3. To put this number into the context of other pump-probe diffraction literature, in semi-metallic 1T-$TiSe_2$, at an absorbed energy density of 1.6×10^{-7} mJ/μm^3, the sample remains intact with no damage[6,7]. Even though the absorbed energy density of LLTO is higher, we note that the value is within a factor of 3 and is within a reasonable expectation of sample-to-sample variation. Note that the value of LLTO is also an upper limit set by the fact that we are assuming a perfectly flat grain.

Supplementary Note 6. Simulation of statistical lattice responses

As mentioned in the main text, the simulation was carried out assuming that each pump-induced lattice response follows the functional form of Eq. (1) and the statistical variation of all shots was collated following the experimental protocol in Fig. 2a. In this section, we will justify the choice of phenomenological parameters in Eq. (1) used in the simulation, where simulation trials with different parameter choices are summarized in Suppl. Table 1 for 100 scans in each trial.

Probability distribution of I_0

The simulations were performed as follows. As discussed in the main text, the initial photoinduced response I_0 has a probability p_0 of changing to a new random value after each pump shot. When I_0 changes, it is drawn from a Gaussian distribution centered around $I_{0,\text{avg}}$, with standard deviation of $0.5 I_{0,\text{avg}}$. Here, $I_{0,\text{avg}}$ is taken from the fitted experimental value of I_0 across all runs (see the solid fitting curve in Fig. 3a). For brevity, we denote this random distribution as $I_0 \sim |G(1, 0.5)|$, where the absolute value ensures that I_0 is non-negative (i.e., lattice expansion). The choice of the 0.5 pre-factor in the standard deviation of the Gaussian distribution is not critical, and it was selected to match the extent of

Suppl. Table 1. A summary of different simulation trials with different parameter choices, where 100 individual scans were simulated and analyzed equivalently to the simulations done in the main text. This table shows how the two parameters τ and I_∞ change when I_0 changes. The result also shows that to recreate the observed trends in the data, indicated by τ_{avg}, τ_{sd}, and ξ, one must set τ to be negatively correlated with I_0 with a steep slope, as indicated by trials 4, 5, 6, 7, and 8. Trial 7 uses the same definitions as the simulation shown in the main text but is simulated over 100 scans (see Suppl. Fig. 10). See text in Supplementary Note 6 for a more detailed description of the simulation procedures.

Trial #	1	2	3	4	5	6	7	8
I_∞ (Å)	$0.2I_0$	$0.2I_{0,\text{avg}}$	$\lvert G(0.2, 0.04)\rvert$	$\lvert G(0.2, 0.04)\rvert$	$0.2I_{0,\text{avg}}$	$0.2I_0$	$0.2I_0$	$0.2I_0$
τ (µs)	τ_0	τ_0	τ_0	Eq. (S9)	Eq. (S9)	Eq. (S10) $\beta = 27, \gamma = 5$	Eq. (S9)	Eq. (S10) $\beta = 30, \gamma = 8$
τ_{avg} (µs)	21.5	19.4	19.0	20.2	17.6	23.2	18.4	20.5
τ_{sd} (µs)	21.5	21.6	21.5	11.7	13.1	17.1	9.9	14.0
ξ (shots)	1410	1560	1480	1370	1450	1450	1470	1420

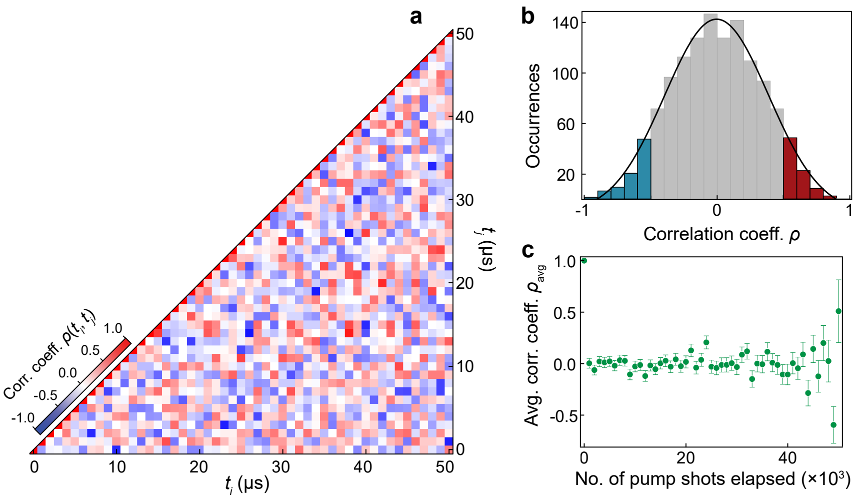

Suppl. Fig. 7. A control simulation when I_0 is random for every pump shot, i.e., $p_0 = 1$. As expected, the correlation matrix in panel **a** indicates random fluctuations of the correlation values except the diagonal line of $t_i = t_j$. These random fluctuations are further verified by the histogram in panel **b**, which is symmetrically distributed around zero. Unlike Fig. 4d,h, a correlation length ξ cannot be extracted in panel **c** since $\rho_{\text{avg}} \approx 0$ as long as $t_i \neq t_j$. Error bars in **c** denote the standard error of ρ_{avg} over 10 scans and they become larger by a factor of $M_{|i-j|}^{-1/2}$ where $M_{|i-j|}$ is the descending number of entries away from $t_i = t_j$ in **a**.

fluctuations present in the correlation matrices in Fig. 4b,f.

We verified that none of the parameters makes a significant contribution to the correlation length ξ except p_0, which scales as $p_0 \sim 1/\xi$. As a control simulation, we computed the correlation matrix when $p_0 = 1$, i.e., the value of I_0 changes after each pump pulse

was delivered to the sample (Suppl. Fig. 7). As expected, there are no correlation patterns outside the diagonal of $t_i = t_j$. This $p_0 = 1$ case would correspond to a scenario where the stochastic lattice response is caused by extrinsic fluctuations such as the variations of the laser pump fluence from shot to shot. Hence, the fact that we see a clear indication of correlated stochastic dynamics in the experimental data (c.f. Figs. 4d and 7c) indicates that the observed variations most likely originate from the random response in the micro-grain itself.

Anti-correlated τ and I_0

As discussed in the main text, the system recovery time τ should be anti-correlated with I_0 in order to reproduce the temporal evolution of $c_{\rm sd}(t)$, whose relaxation time $\tau_{\rm sd}$ is markedly shorter than $\tau_{\rm avg}$, the relaxation time of the averaged evolution $c_{\rm avg}$.

Supplementary Table 1 justifies the relation between τ and I_0. If τ is independent from I_0 and is set to a canonical value $\tau_0 = 22$ µs (taken to be close to experimental $\tau_{\rm avg}$), then $\tau_{\rm sd}$ is nearly identical to $\tau_{\rm avg}$ in the simulation (see trials 1–3 in Suppl. Table 1). If τ is anti-correlated with I_0, we find $\tau_{\rm sd} < \tau_{\rm avg}$ as observed in our experiments (see trials 4–8). The functional form of $\tau(I_0)$ is not critical, and Fig. 4e–h in the main text is simulated based on the following form

$$\tau = \tau_0 \left[1 - \mathrm{erf}\left(\frac{I_0/I_{0,\rm avg} - 1}{\sigma}\right)\right]. \tag{S9}$$

The error function is introduced to avoid instances where τ could become negative, which is an unphysical situation. Here, σ controls the slope of the error function near $I_0 = I_{0,\rm avg}$. To demonstrate that the functional relationship between τ and I_0 is not critical, we also performed simulations based on a linear relationship,

$$\tau = \beta - \gamma I_0/I_{0,\rm avg}, \tag{S10}$$

and trials 6 and 8 in Suppl. Table 1 show the results of such simulations. In both Eqs. (S9) and (S10), τ is linearly proportional to I_0 when $I_0 \sim I_{0,\rm avg}$. We found that a steeper slope of this linear dependence typically results in a lower value of $\tau_{\rm sd}$, and a value of $\sigma = 2.57$

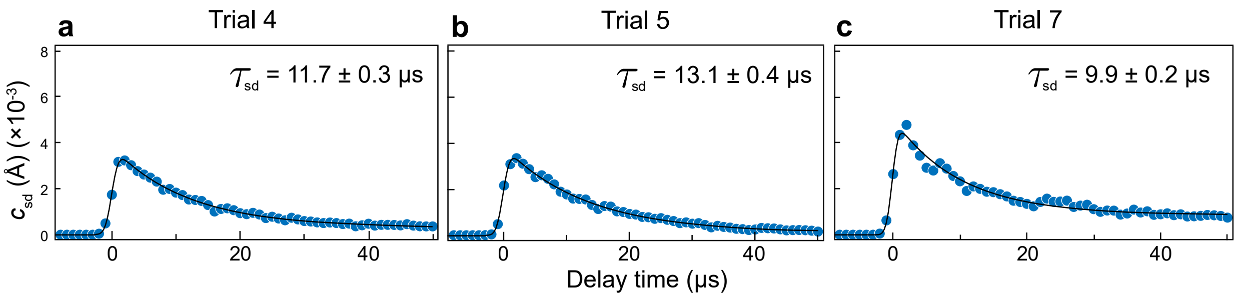

Suppl. Fig. 8. Standard deviation of the lattice c parameter (c_{sd}) of 100 simulated scans for three trials to show the difference I_∞ makes in the later delay times of the photoinduced dynamics. Panel **a** shows trial 4 from Suppl. Table 1 where I_∞ is drawn randomly from a Gaussian distribution. Panel **b** shows trial 5 where I_∞ is always constant, and panel **c** shows the simulation where I_∞ is linearly proportional to I_0. The standard deviation c_{sd} at late times in **c** is higher than **a** or **b**, so trial 7 more accurately reproduces the raw data in Fig. 3b.

was chosen in Eq. (S9) to match the experimental value of τ_{sd} in Fig. 3b.

Choice of I_∞

When I_0 is updated after a pump laser shot with probability p_0, I_∞ is also updated. Following the physical scenario that the long-term quasi-equilibrium lattice state should be positively correlated with the initial change of the lattice right after photoexcitation, we defined I_∞ to be linearly and positively proportional to I_0 to the lowest order. The exact functional form used in the simulation is $I_\infty = 0.2I_0$, but this choice is not critical. As shown in Suppl. Table 1, I_∞ can also be randomly drawn from a Gaussian distribution or remain a constant to reproduce the two main observations in the data, namely, $\tau_{\mathrm{sd}} < \tau_{\mathrm{avg}}$ and $\xi \approx 1,500$ shots, provided that the conditions for τ have been met as discussed in the previous section. Figure 8 shows these differing scenarios in the definition of I_∞ to compare the temporal evolution of σ_{sd} from selected trials in Suppl. Table 1. The parameters in trial 7 were used in the main text as Suppl. Fig. 8c shows that the long tail of the simulated c_{sd} matches better with the experimental data.

Choice of t_0 and w

From a physical standpoint, fluctuations in t_0 and w are not expected to significantly contribute to the temporal evolution of the lattice over the microsecond time scale. More

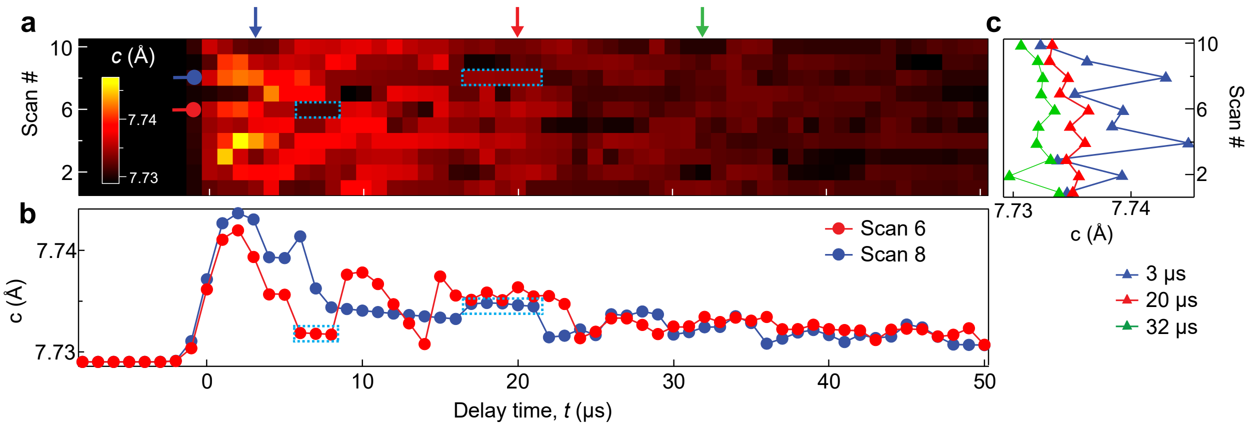

Suppl. Fig. 9. Simulated data in the same format as presented in Fig. 2c–e of the main text. Key features from the experiments are reproduced: (i) streaks followed by discontinuities are clearly visible in the time traces (dashed boxe in panels **a**,**b**), and (ii) time slices show that the variance near time zero is much larger compared to that at later delay times (panel **c**).

specifically, t_0 is affected by the timing uncertainty of pump-probe temporal overlap, which has an upper bound of the probe pulse duration of 100 ps. w is determined by the timescale of the initial lattice expansion, which occurs well below the step size of 1 μs, as demonstrated by a single-step initial jump of the lattice parameter in our experiment (Fig. 3a). Therefore, these parameters were set to constant values during the simulation: $w = 2$ μs and $t_0 = 0$ μs.

Additional analysis of the simulations

As an additional validation of the simulation procedure, we compared the generated temporal evolutions to those measured in the experiment (c.f. Figs. 2c,d and 9a,b), where discontinuities of the lattice response in between streaks of similar values are clearly observed. Similar to the experimental data, variation of the lattice response at a time delay right after time zero is also much larger than at a later delay (c.f. Figs. 2e and 9c).

In Fig. 4e–h in the main text, the simulation was carried out for 10 scans to recreate the experimental condition. Using the same set of parameters, we also extended the scan number to 100 (trial 7 in Suppl. Table 1), and the results are shown in Suppl. Fig. 10. The main conclusions about $\tau_{\mathrm{sd}} < \tau_{\mathrm{avg}}$ and a finite value of ξ remain robust, and the random fluctuations present in the correlation matrix (c.f. Figs. 4b and 10c) are reduced. Importantly, the positive correlations near the diagonal of the correlation matrix ($t_i \approx t_j$) remain in Suppl. Fig. 10c, highlighting the effectiveness of our methodology in uncovering

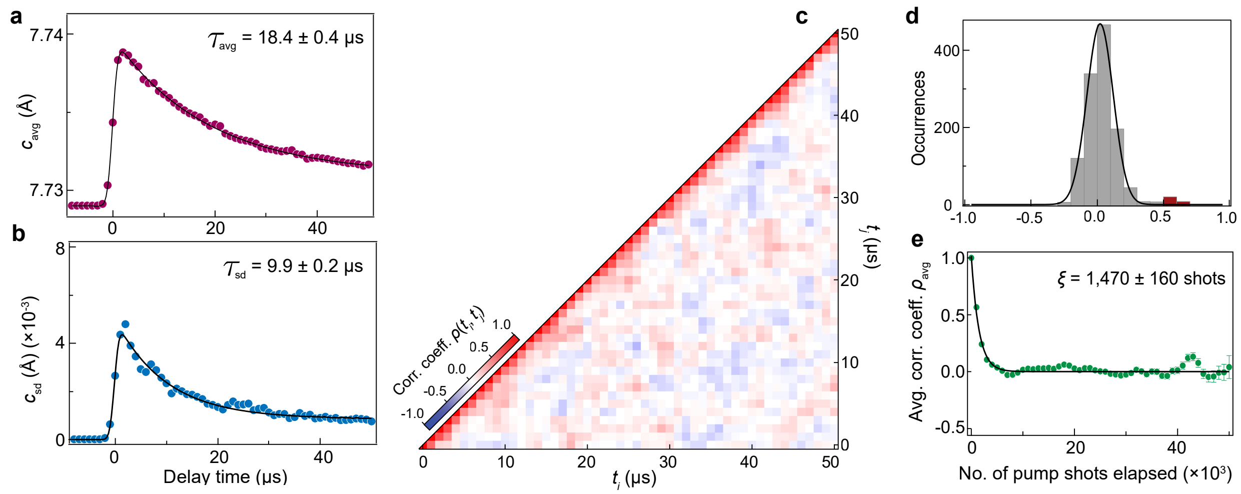

Suppl. Fig. 10. Simulated data from trial 7 in Suppl. Table 1 over 100 scans instead of 10 scans as done in Fig. 3c,d and Fig. 4e–h, where panel **a** is the average c axis parameter and panel **b** is the standard deviation. This figure highlights the robustness of the conclusions about $\tau_{\mathrm{sd}} < \tau_{\mathrm{avg}}$ and a finite ξ value against different scan averages. It is worth noting the small shoulder in the histogram (colored red in panel **d**), which originates from the positive correlations when $t_i \approx t_j$ in panel **c**. Error bars in **e** denote the standard error of ρ_{avg} over 100 scans and they become larger by a factor of $M_{|i-j|}^{-1/2}$ where $M_{|i-j|}$ is the descending number of entries away from $t_i = t_j$ in **c**.

correlated dynamics in a nonequilibrium system.

Supplementary Note 7. Estimate of lattice temperature after photoexcitation

In the main text, we mentioned a transient increase of the micro-grain lattice temperature by 320 K following photoexcitation. Here, we explained how we computed this number, along with error bars. At the microsecond timescale, the system reaches a quasi-equilibrium state where the lattice expands as a a result of laser-induced heating. Hence, one way to estimate the transient lattice temperature in this regime is by benchmarking the measured c-axis elongation against equilibrium thermal expansion coefficients[8], which were tabulated previously[9] and are approximately 10^{-5} K^{-1} at and above room temperature. We note that the thermal expansion coefficient is bounded by approximately $(1 \pm 0.4) \times 10^{-5}$ K^{-1}. With this in mind, we take the maximum change of the c value in Fig. 2c, where $\Delta c/c = 0.32\%$, and we have a transient temperature increase of $\Delta T = 320 \pm 100$ K, where the error bar corresponds to the limits of the thermal expansion coefficient. This error seems large, but results in the reported error of ± 0.1 eV for the energy barrier due to the exponential sup-

pression in the Boltzmann distribution during error propagation.

Supplementary References

[1] L. Zhang, X. Zhang, G. Tian, Q. Zhang, M. Knapp, H. Ehrenberg, G. Chen, Z. Shen, G. Yang, L. Gu, and F. Du, Lithium lanthanum titanate perovskite as an anode for lithium ion batteries, Nat. Commun. **11**, 3490 (2020).

[2] H. Geng, A. Mei, C. Dong, Y. Lin, and C. Nan, Investigation of structure and electrical properties of $Li_{0.5}La_{0.5}TiO_3$ ceramics via microwave sintering, J. Alloys Compd. **481**, 555 (2009).

[3] S. Matzen, L. Guillemot, T. Maroutian, S. K. K. Patel, H. Wen, A. D. DiChiara, G. Agnus, O. G. Shpyrko, E. E. Fullerton, D. Ravelosona, P. Lecoeur, and R. Kukreja, Tuning Ultrafast Photoinduced Strain in Ferroelectric-Based Devices, Advanced Electronic Materials **5**, 1800709 (2019).

[4] A. Zong, Q. Zhang, F. Zhou, Y. Su, K. Hwangbo, X. Shen, Q. Jiang, H. Liu, T. E. Gage, D. A. Walko, *et al.*, Spin-mediated shear oscillators in a van der waals antiferromagnet, Nature **620**, 988 (2023).

[5] A. Chouiekh, A. Tahiri, N. E. H. Bouftila, A. Nfissi, L. Bih, A. Faik, T.-d. Lamcharfi, Y. Ababou, A. Rjeb, and M. Naji, Experimental and DFT analysis of structural, optical, and electrical properties of $Li_{3x}La_{2/3-x}TiO_3$ ($3x = 0.1$, 0.3 and 0.5) solid electrolyte, Ceramics International **49**, 25920 (2023).

[6] Y. Cheng, A. Zong, J. Li, W. Xia, S. Duan, W. Zhao, Y. Li, F. Qi, J. Wu, L. Zhao, *et al.*, Light-induced dimension crossover dictated by excitonic correlations, Nat. Commun. **13**, 963 (2022).

[7] Y. Cheng, A. Zong, L. Wu, Q. Meng, W. Xia, F. Qi, P. Zhu, X. Zou, T. Jiang, Y. Guo, J. van Wezel, A. Kogar, M. W. Zuerch, J. Zhang, Y. Zhu, and D. Xiang, Ultrafast formation of topological defects in a two-dimensional charge density wave, Nat. Phys. **20**, 54 (2024).

[8] F. Zhou, K. Hwangbo, Q. Zhang, C. Wang, L. Shen, J. Zhang, Q. Jiang, A. Zong, Y. Su, M. Zajac, Y. Ahn, D. A. Walko, R. D. Schaller, J.-h. Chu, N. Gedik, X. Xu, D. Xiao, and H. Wen, Dynamical criticality of spin-shear coupling in van der Waals antiferromagnets, Nat. Commun. **13**, 6598 (2022).

[9] M. Bertrand, S. Rousselot, D. Aymé-Perrot, and M. Dollé, Compatibility assessment of solid ceramic electrolytes and active materials based on thermal dilatation for the development of solid-state batteries, Mater. Adv. **2**, 2989 (2021).
