# Supplementary material for: Photoinduced correlations in stochastic dynamics of a solid-state ionic conductor
Source: Nat Commun. 2026 May 15;17:6552. doi: 10.1038/s41467-026-72663-7 (PMC13381525; doi:10.1038/s41467-026-72663-7)
Supplement: Supplementary file 2 — Transparent Peer Review file [file 41467_2026_72663_MOESM2_ESM.pdf]

nature portfolio

# Peer Review File

# Photoinduced correlations in stochastic dynamics of a solid-state ionic conductor

Corresponding Author: Professor Michael Zuerch

**This file contains all reviewer reports in order by version, followed by all author rebuttals in order by version.**

Version 0:

Reviewer comments:

Reviewer #1

(Remarks to the Author)
The manuscript presents an investigation into the stochastic dynamics of a solid-state ionic conductor (LLTO) using time-resolved X-ray micro-diffraction. The main achievement of this work is the identification and characterization of fluctuations in the lattice response of LLTO following photoexcitation by ultrashort laser pulses. By employing various statistical methods, the study reveals correlations in the lattice dynamics, offering insights into the underlying physical processes that govern the material's behavior. Specifically, the authors propose that lithium-ion diffusion in the material leads to switching between dynamical pathways, resulting in the stochastic response after photoexcitation.

The study utilizes state-of-the-art time-resolved X-ray micro-diffraction techniques to collect high-quality data, which is presented clearly and effectively in the figures. The experimental setup and methodology are robust, reflecting a high standard of scientific rigor. The data analysis is comprehensive and detailed; the results are both novel and original. It has to be noted, though, that the study investigates microseconds to milliseconds dynamics despite the significantly higher time resolution provided by the laser pump and x-ray probe.

One of the most significant findings of the study is the demonstration of how the stochastic behavior of the sample's response to photoexcitation is captured in the diffraction data. The authors can clearly attribute the “noise” to sample fluctuations and distinguish it from any instrumental instabilities (e.g. source, excitation, detection), making the study a showcase example for the community for studying stochastic pathways in a response to photoexcitation.

However, I have questions and concerns about the broad applicability and impact of the method. The statistical analysis has two separate parts. In the first one, the authors simply analyze the standard deviation of the noise in the data. They find that an increased noise level after photoexcitation must be related to a fluctuating response of the sample after excluding all other sources of noise. While this approach works impressively for the synchrotron data presented, I have doubts that this distinction can be made for experiments at FEL or HHG sources (or other laser-based sources), where such time-resolved experiments are often conducted. These sources are characterized by intense intrinsic fluctuations of many source parameters. In addition, the standard deviation is a very unspecific statistical quantity, and it remains unclear from the manuscript how the findings presented in Fig. 3 can be leveraged to build a physical model and obtain quantitative measures of the actual sample fluctuations.

The correlation analysis in the second part is more powerful in this respect. It basically applies correlation methods that are commonly used in, e.g., x-ray photon correlation spectroscopy (XPCS), although the manuscript does not explicitly mention this. As these are well-established concepts, I find Fig. 4 somewhat inflated. It basically boils down to panel d showing the temporal autocorrelation. I do not see any value in showing the two-time correlation functon(panel b) when the dynamics is temporally homogenous. Also, the value of the insets in panel a and the histogram in panel c is unclear. While this kind of correlation analysis indeed provides some interesting insights into the underlying processes in the LLTO sample, I have strong concerns about the method’s general applicability as it strongly depends on the presence of some degree of persistence or memory over a certain number of shots in the sample. The sample must switch between states or phases on the lab time scale (here, milliseconds) rather than on the time scale of the photo-response (here, microseconds but potentially picoseconds). In my view, this dramatically limits the application spectrum of the method (i) in terms of sample systems and (ii) to slow stochastic dynamics that can potentially be investigated by alternative, simpler methods. Most importantly, this correlation analysis will not work in the case where the dynamics show truly “random fluctuations” with

“shot-to-shot variations” as it is advertised throughout the manuscript. All references provided as examples of a fluctuating sample response (Ref 11-13) fall into this category and could not be analyzed with the correlation method proposed by the authors.

The authors also provide a “simulation” of the stochasticity in the data. However, this simulation is only an MC-like implementation using the statistical properties of the experimental data and does not contain any physical model. It is evident that such a model must recreate similar data with the same statistical signatures. I do not understand what can be learned from this model.

At some points in the manuscript, the language is too exaggerated. The authors frequently use the term “hidden correlations”, while a simple autocorrelation is sufficient to unveil the correlations. The term “disordered fluctuations” is a pleonasm. I am also not convinced that the method presented deserves its own name (“nonequilibrium noise correlation spectroscopy”) as all experimental and statistical tools are well-established, and also their combination is not particularly original. The terminology is even scientifically inaccurate, as the “correlation” analysis can only be applied under quasi-equilibrium conditions. Otherwise, the Arrhenius assumption would not work. In line 230, the authors are “Inspired by the excellent fit of Eq. (1) to the averaged response in Fig. 3a”. However, this is a standard generic model with many fit parameters, which is able to fit almost any time-resolved data of this kind. “[…] probing stochastic processes” (line 27) on ultrafast time scales is certainly challenging but not puzzling and, thus, not a “conundrum.”

In summary, the authors have not convincingly explained how their method can be applied to study photoinduced processes with “random fluctuations,” which manifest in the data as “shot-to-shot variations” beyond simply detecting the existence of these fluctuations. The sample case presented is undoubtedly interesting, but it remains specific. I see substantial limitations of the application of the method in terms of X-ray sources, sample systems, and time scales of the dynamics, which directly limit the impact of the work. Major revision of the manuscript is required to address these aspects.

Reviewer #2

(Remarks to the Author)
I have co-reviewed the manuscript with one of the listed reviewers.

Reviewer #3

(Remarks to the Author)
The manuscript “Hidden correlations in stochastic photoinduced dynamics of a solid-state ionic conductor” presents a method termed “nonequilibrium noise correlation spectroscopy”, which describes a statistical framework for extracting shot-to-shot correlations in pump–probe experiments. Applied to the transition metal compound Li0.5La0.5TiO3 and using synchrotron-based pump-probe X-ray diffraction, the study reveals correlated lattice responses with a characteristic “correlation length” of approximately 1,500 laser shots, corresponding to a switching energy of ~0.4 eV.

The work is relevant as it introduces a new statistical approach to extract correlations between individual pump shots in a stroboscopic X-ray pump–probe experiment. Unlike conventional methods that average out fluctuations or focus on single delay times, this technique quantifies shot-to-shot variations using synchrotron measurements with high stability and photon flux. The work meets the standards expected for publication in a journal such as Nature Communication. The method is described comprehensively, such that it should be possible to reproduce the results following the sample preparation, experimental measurement and statistical analysis description.

The manuscript is of good quality, the fit parameters used in the equations and simulations are clearly explained, and the influence of possible external noise sources is addressed. As the work is sound and concerns an important question, we feel that it will be suitable for publication in Nature Communications if the authors address the following questions :

1. The manuscript presents multiple, distinct time scales—microseconds, seconds, and minutes—that are somewhat confusing. In both the “Time-resolved X-ray diffraction” section and the caption of Fig. 2, the same experimental procedure is described, but the relationship between these time scales is not entirely clear from neither of them. Only later, when the correlation analysis in Fig. 4 is discussed, it is stated that 1 µs corresponds to 1000 pump shots. Is our understanding correct that Fig. 2c,d show delay times $t_{1 \ldots 59}$, each spaced by 1 µs, but that there are 1000 pump shots (1 s) between each consecutive delay time point $t_i$ and $t_{i+1}$?
The authors should clarify this in the main text where the results of Fig. 2 are discussed, and revise the caption of Fig. 2 to better explain the connection between the various time scales. We suggest also labeling the axes in Fig. 2c,d more clearly (e.g. explicitly marking $t_i$ and noting n = 1000 shots), which would make the figure more intuitive and more clearly linked to the schematic in Fig. 2a.

2. The authors should add a few sentences explaining the possible physical origin of the correlations (length) observed between different delay times.

3. It is stated that, because only one Bragg peak (not a ring) is observed, the signal must originate from a single grain. Was it explicitly verified that no ring appears in any of the individual shots of each scan? If so, how good is the shot-to-shot position stability of this Bragg peak?

4. How can the authors be sure that the signal between different shots is coming from the same grain?

5. Possibly related to point 4, in Supplementary Note 2 the authors mention using a long-term drift correction. Given the presence of several distinct time scales in this experiment, they should clarify what is meant by "long." Specifically, how much drift correction is needed for the pre-time-zero averages, and over what time frame and how does this relate to the different time scales (see question 1). Does the extracted “correlation length” agree with and without drift correction?

Reviewer #4

(Remarks to the Author)
I co-reviewed this manuscript with one of the reviewers who provided the listed reports. This is part of the Nature Communications initiative to facilitate training in peer review and to provide appropriate recognition for Early Career Researchers who co-review manuscripts.

Version 1:

Reviewer comments:

Reviewer #1

(Remarks to the Author)
I appreciate the effort the authors have put into revising the manuscript and providing an extensive rebuttal. The high quality of the synchrotron micro-diffraction data is unquestionable, and the observation of stochastic photoinduced pathways in LLTO remains both original and of scientific impact within the ultrafast science community. Figures 1 and 2 are exceptionally clear and effectively guide the reader through the experimental scheme and the observed phenomenology.

That said, even after revision, I find several aspects of the presentation stylistically inflated. Many of the core observations are conceptually straightforward, yet they are described in a lengthy and overly elaborate manner that does not improve clarity. This issue was not resolved in the revised version and, if anything, the rebuttal reinforced my impression that simple points are being presented with unnecessary complexity. The expanded discussion around the standard deviation is a good example. Although the rebuttal attempts to justify its importance, the manuscript itself never integrates this quantity into the physical discussion or uses it to advance the interpretation. As a result, it remains unclear why this analysis is needed or how it contributes to the main conclusions. Similarly, my concerns about Fig. 4 remain. The extended two-time correlation matrix and its accompanying subfigures still feel disproportionate to the insight they provide, especially given the simulation's ineffectiveness (see following comment).

Simulation
The value of the simulation remains unclear. As currently implemented, it is essentially tautological: the authors observe an exponential decay of correlations, simulate a generic process that is explicitly constructed to exhibit the same exponential decay, and then conclude that the simulation “reproduces” the experimental behavior. This does not provide additional physical insight, nor does it validate the proposed mechanism. In fact, the observed dynamics correspond more closely to a simple telegraph-noise (two-state switching) model with a characteristic lifetime rather than to a genuine random walk. I would encourage the authors either to substantially reframe the purpose of the simulation as purely illustrative or to develop a model that discriminates between alternative physical scenarios rather than reproducing the statistics by construction.

Applicability of the Method
The applicability of the proposed method still appears overstated. Its usefulness hinges on long-lived shot-to-shot persistence over thousands of pump pulses, which is a highly unusual condition in ultrafast pump-probe experiments. As such, the method cannot reveal genuinely random fluctuations, e.g., those near a critical point or governed by fast, nondeterministic quasiparticle dynamics, despite the phrasing in the introduction suggesting otherwise.
In their rebuttal, the authors list several examples of metastable or switching phenomena from the literature. These systems do indeed exhibit persistence in various forms, but they are not integrated into the manuscript. In addition, they do not clearly demonstrate the specific type of long-lived, shot-correlated stochastic dynamics required for the present method. Some of the cited works probe dynamics that are directly observable in single-shot measurements or rely on deterministic photoinduced switching rather than hidden correlations. Concerning the references currently cited in the manuscript: The authors agreed that the persistence required was not reported or implied in these works. The claim that “it could still be the case” that they do so is speculative and unsupported.

Relation to XPCS
I appreciate that the revised manuscript now situates the method more explicitly within the context of correlation analysis techniques developed for XPCS. However, I would caution against drawing too close an analogy. A central strength of XPCS is its interference-based sensitivity, which allows extremely small structural fluctuations to be detected over relatively large illuminated volumes. The present method, by contrast, relies on micro-focused single-grain diffraction to achieve sufficient local sensitivity for observing stochastic switching. While this is well-suited to the current synchrotron experiment, it also introduces practical constraints, particularly in FEL settings, where achieving micron-scale focusing with adequate stability is technically challenging. Moreover, the high fluence of tightly focused FEL pulses raises significant concerns about radiation damage, which may preclude the repeated, persistent probing of a single micrograin that this method requires. Thus, the approach is complementary to XPCS but should not be presented as a streamlined substitute; its

applicability remains inherently limited by the need for highly localized, low-damage probes and exceptional stability.

In summary, while the experimental data are of high quality and the observation of stochastic photoinduced pathways is genuinely interesting, the manuscript in its current form does not meet the standards for publication in Nature Communications. The scope and applicability of the method are overstated, key arguments remain speculative or insufficiently justified, and several aspects of the presentation obscure rather than clarify the underlying physics. Substantial revision would be required to address these issues. I therefore recommend major revision before the manuscript can be considered for publication.

Reviewer #3

(Remarks to the Author)
The authors have thoroughly addressed the questions raised by us and the other reviewers, and in the revised manuscript provide additional information that improves the overall clarity.

One remaining point concerns our previously raised question 5 regarding long-term drifts. The authors explain the origin and explicitly stated in their response that the drift correction is $1\times10^{-3}$ Å. Given that this value is small and not visible in the before and after correction plot comparison, the authors should also include it in Supplementary Section 2.

Apart from this minor addition, we believe the manuscript is now suitable for publication in Nature Communications.

In addition, I have been asked to comment on the concerns of reviewer #1 from the current review round.

Reviewer #1 also acknowledges the high quality of the synchrotron micro-diffraction data, as well as the originality and potential impact of the results and the technique for the ultrafast condensed matter physics community.

In addition, reviewer #1 raises some concerns regarding the presentation of the results which I support to a certain extent. But in my opinion, this is principally a matter of style and wording rather than content. Therefore, I still feel that the manuscript should be considered for publication. I suggest minor revisions where the authors should simplify and streamline the discussion and generally adapt / tone down the phrasing in the spirit of reviewer #1's comments. Finally, I support reviewer #1's request that also challenges in the applicability of the method are discussed more openly, e.g., concerning beam stability and sample damage at X-ray FELs.

With those revisions implemented I trust that the manuscript will meet the standards for publication in Nature Communications.

Reviewer #4

(Remarks to the Author)
I co-reviewed this manuscript with one of the reviewers who provided the listed reports. This is part of the Nature Communications initiative to facilitate training in peer review and to provide appropriate recognition for Early Career Researchers who co-review manuscripts.

Version 2:

Reviewer comments:

Reviewer #1

(Remarks to the Author)
I appreciate the substantial effort the authors have invested in revising the manuscript and addressing the concerns raised in the previous review rounds. In particular, the discussion of the method's applicability has been significantly improved and is now presented in a more balanced and carefully qualified manner. The authors have clearly taken the earlier comments seriously, and the manuscript has benefited from these revisions. Our earlier disagreement regarding the presentation remains a matter of style.
However, I remain unconvinced about the added value of the simulation. Its outcome is largely predictable from the experimentally observed exponential decay of correlations. The first claimed insight, the anti-correlation between I_0 and tau, already follows from the analysis of the experimental data in Fig. 3. The simulation does not demonstrate that this anti-correlation is uniquely required, as no case is shown where removing or modifying this assumption leads to a breakdown of the model. Similarly, the extracted switching probability p_0 essentially reflects the measured correlation length xi and can simply be estimated as p_0 = 1/xi = 0.07%, without the need for a full MC implementation, which yielded p_0 = 0.09%. This minor deviation of p_0 from the estimate depends on model assumptions (choice of probability distribution and imposed anti-correlation), which are not independently validated.
The issue is therefore not that the simulation is technically incorrect, but that its role is presented in a misleading way (lines 256-264). The manuscript emphasizes that the model "recreated" the two-time correlation matrix, histogram, streak structure, and correlation length, and that the agreement with experiment is nearly indistinguishable. However, this agreement is inherent to the way the model is mathematically constructed and tuned to reproduce the observed correlation decay. As the simulation misses any physical layer, it should be described as illustrative or a consistency check, rather than as providing

independent explanatory insight. Still, I would consider this a minor issue remaining.

**Open Access** This Peer Review File is licensed under a Creative Commons Attribution 4.0 International License, which permits use, sharing, adaptation, distribution and reproduction in any medium or format, as long as you give appropriate credit to the original author(s) and the source, provide a link to the Creative Commons license, and indicate if changes were made.
In cases where reviewers are anonymous, credit should be given to 'Anonymous Referee' and the source.
The images or other third party material in this Peer Review File are included in the article's Creative Commons license, unless indicated otherwise in a credit line to the material. If material is not included in the article's Creative Commons license and your intended use is not permitted by statutory regulation or exceeds the permitted use, you will need to obtain permission directly from the copyright holder.
To view a copy of this license, visit https://creativecommons.org/licenses/by/4.0/

**Response to Reviewers for the Manuscript Titled**

*"Photoinduced correlations in stochastic dynamics of a solid-state ionic conductor"*

*(new title, previously "Hidden correlations in stochastic dynamics of a solid-state ionic conductor")*

We thank all referees for their careful review and constructive feedback, which have substantially helped us improve the quality of this work. Below, we provide a point-by-point response, where the referees' comments are shown in **black**, our responses are shown in **teal**, and manuscript amendments are shown in **red**.

# Reviewer #1

**Rev. 1 Comment 1:** The manuscript presents an investigation into the stochastic dynamics of a solid-state ionic conductor (LLTO) using time-resolved X-ray micro-diffraction. The main achievement of this work is the identification and characterization of fluctuations in the lattice response of LLTO following photoexcitation by ultrashort laser pulses. By employing various statistical methods, the study reveals correlations in the lattice dynamics, offering insights into the underlying physical processes that govern the material's behavior. Specifically, the authors propose that lithium-ion diffusion in the material leads to switching between dynamical pathways, resulting in the stochastic response after photoexcitation.

The study utilizes state-of-the-art time-resolved X-ray micro-diffraction techniques to collect high-quality data, which is presented clearly and effectively in the figures. The experimental setup and methodology are robust, reflecting a high standard of scientific rigor. The data analysis is comprehensive and detailed; the results are both novel and original. It has to be noted, though, that the study investigates microseconds to milliseconds dynamics despite the significantly higher time resolution provided by the laser pump and x-ray probe.

One of the most significant findings of the study is the demonstration of how the stochastic behavior of the sample's response to photoexcitation is captured in the diffraction data. The authors can clearly attribute the "noise" to sample fluctuations and distinguish it from any instrumental instabilities (e.g. source, excitation, detection), making the study a showcase example for the community for studying stochastic pathways in a response to photoexcitation.

We thank the Reviewer for taking their time to read our manuscript in detail and we recognize the high-level summary of the key findings of the paper. We especially appreciate the positive notes on the data quality and methodology presented in our study. Given the feedback, we

agree that our manuscript will benefit from changes which we will address in the following response.

The key to our method does indeed rely on the high signal-to-noise ratio of our source. Specifically, the sample fluctuations must be distinguishable from instrumental sources as we discuss in more detail below.

**Rev. 1 Comment 2:** However, I have questions and concerns about the broad applicability and impact of the method. The statistical analysis has two separate parts. In the first one, the authors simply analyze the standard deviation of the noise in the data. They find that an increased noise level after photoexcitation must be related to a fluctuating response of the sample after excluding all other sources of noise. While this approach works impressively for the synchrotron data presented, I have doubts that this distinction can be made for experiments at FEL or HHG sources (or other laser-based sources), where such time-resolved experiments are often conducted. These sources are characterized by intense intrinsic fluctuations of many source parameters.

While we appreciate the accurate summary and compliment on the statistics of the data presented, we aim to address the central concern about the broad applicability of the method. The key to this method is separating sample fluctuations from the source (here, the synchrotron), which can be done using many different sources.

The Reviewer brings up a valid concern about the applicability of this method using an FEL source, considering as a previous study demonstrates dynamically evolving correlations between pulse events at the European XFEL. [Guest, Trey W., et al. *IUCrJ*. **10.6** (2023): 708-719]. However, the same study simulates these correlations using a phenomenological model. Using this model, the authors suggest that in cases where the pulse intensity of the source can be recorded simultaneously during diffraction experiments, the development of filtering algorithms that consider pulse-to-pulse correlations may separate these underlying fluctuations from those of interest. [Rose, M., et al. *IUCrJ*. **5**, (2018): 727–736]. Despite this, FEL sources can still reveal stochastic processes as demonstrated in [Fletcher, Luke B., et al. *Sci. Rep.* **14.1** (2024): 17283] where a single-shot pump X-ray probe XPCS reveals disordered XRD patterns on random stacking sequences of supercooled hydrogen.

The Reviewer also mentions high harmonic generation, which also could be challenging considering pulse-to-pulse energy fluctuations are larger in highly nonlinear regimes. However, there have been recent advances in self-referencing of HHG sources which aim to minimize background signals and suppress noise [Volkov, Mikhail, et al. *Opt. Express*. **27.6**

(2019): 7886-7895], which could greatly expand the possibility of observing sample-intrinsic fluctuations.

One example of probing stochastic processes using a tabletop laser source is in [Weiss, M. A., et al. *Nat. Commun*. **14.1** (2023): 7651]. Although not a direct observation of spin switching, this study used two femtosecond pulses transmitted through $Sm_{0.7}Er_{0.3}FeO_3$ and extracted the autocorrelation function from the polarization changes of the two sources at varying time delays after sample transmission. Such correlations are related to transient magnetic fluctuations which occur on hundreds of picoseconds, and near the critical temperature. This study could be expanded upon by using magnetic-sensitive probes to separate orbital and spin contributions to magnetization such as X-ray magnetic circular dichroism (XMCD) [Borchert, Martin, et al. *Optica* **10.4** (2023): 450-455] or probes that couple with magnons whose frequencies are in the terahertz regime [Makihara, Takuma, et al. *Nat. Commun*. **12.1** (2021): 3115]. Therefore, it is evident that sample fluctuations can be distinguished using several sources.

To summarize, although separating out the source noise may not be trivial in certain cases, many improvements are being made to develop more analytical techniques and enhance signal-to-noise to curb these challenges, making our analysis framework broadly applicable to an increasing number of experimental platforms as laser technologies rapidly advance. Also, we note that ultimately what matters is how source fluctuations compare to stochastic fluctuations in the physical process studied, which of course can vary. Our choice of using a synchrotron in this experiment was informed by our interest in studying the lattice/ionic response to photoexcitation in LLTO, but we do not believe the method demonstrated in the manuscript is limited to synchrotrons as outlined above. Although, including a repeated experiment using an FEL or HHG source (or an extensive discussion of the two) would not fundamentally add to this already expansive paper considering the long SI and would diffuse the main message of the paper in connection with the insights into the ionic behavior we convey in our work.

**Rev. 1 Comment 3:** In addition, the standard deviation is a very unspecific statistical quantity, and it remains unclear from the manuscript how the findings presented in Fig. 3 can be leveraged to build a physical model and obtain quantitative measures of the actual sample fluctuations.

We thank the Reviewer for raising concerns about the usage of the standard deviation in this context, and we believe that the standard deviation here could have been better defined. We aim for this quantity to be presented in a very specific manner, specifically, the standard

deviation and average of the lattice parameter in Fig. 3b gives us an idea of the distribution of possible lattice configurations at a given time delay. However, it should be noted that this quantity ($c_{sd}$) is calculated from data that is already averaged (i.e., each datapoint is an accumulated average of approximately 1,000 individual shots). Since we expect laser pulses to modulate the equilibrium conditions of LLTO, it is beneficial in a physical sense to know what the distribution of possible lattice configurations are as a function of time due to a single laser pulse. To be exact, we want to know the standard deviation of the lattice parameter if it were calculated across 10,000 individual shots ($\sigma_{shot}$). To extract this quantity, if one assumes that the response from each pulse event is uncorrelated, or that each pulse event is statistically independent from one another, and arrives at the relationship:

$$c_{sd}^2 = \frac{\sigma_{shot}^2}{n},$$

Where $n$ is the sampling size and $\sigma_{shot}$ is the standard deviation of the lattice parameter response across all shots. However, we did not make this assumption in our manuscript because this relationship breaks down in the event of correlations, which were observed in our experiment over the course of thousands of shots. In the presence of a finite correlation times, the relationship is as follows [Flyvbjerg, Henrik, and Henrik Gordon Petersen. *J. Chem. Phys.* **91.1** (1989): 461]:

$$c_{sd}^2 = \frac{\sigma_{shot}^2}{n} \times \sum_{i,j=0}^{n} \rho_{i,j}$$

Where $\rho_{i,j}$ is the autocorrelation function as indicated by the fit in Fig. 4. Using our parameters ξ = 1,500 shots, and $n$ = 1,000 shots, we find that $c_{sd} \approx 0.85\sigma_{shot}$ therefore, our calculated standard deviation essentially reports the distribution of possible values of the lattice parameter given a single laser shot.

We regret that these aspects were not represented carefully enough in our initial submission. We made the following changes and additions to the manuscript:

**Main text in "Time-resolved X-ray micro-diffraction"**

This scan-to-scan variation has significant physical value in that it is directly proportional to the statistical variation of the lattice response over all individual shots (see Supplementary Note 6 for further details). For independent uncorrelated events, the relationship between these two quantities depends only on *n,* the number of shots at one delay time. However, we turn to our second observation, in which the discontinuities in the time traces are often preceded or succeeded by a streak of similar values, as highlighted in dashed boxes in Fig.

2c,d. The presence of these streaks is unexpected if the stochastic lattice dynamics following each pump pulse are independent of each other. The streaks hence hint at some correlated photoinduced dynamics in LLTO. We will quantify both features of the stochastic response in the statistical analyses below.

**Added "Supplementary Note 6. Shot variation of the *c* lattice parameter"**

As mentioned in the main text, the standard deviation calculation in Fig. 3b ($c_{sd}$) is proportional to the standard deviation as a measure of individual laser shots to the sample ($\sigma_{shot}$). This section will calculate $\sigma_{shot}$ while considering the correlation time obtained from the fit in Fig. 4d.

Since we do not have access to the lattice response after an individual pulse event, $c_{sd}$ is generally referred to as the standard error, where this quantity is calculated over a group of data that is already averaged over 1,000 shots. To extract the true standard deviation ($\sigma_{shot}$), we can use the relationship below where *n* = 1,000 shots is the sample size.

$$c_{sd}^2 = \frac{\sigma_{shot}^2}{n} \times \sum_{i,j=0}^{n} \rho_{i,j}$$

$\rho_{i,j}$ is the correlation function presented as the fit in Fig. 4d that arises from correlated pulse events. For $n = 1{,}000$ shots and $\xi = 1{,}500$ shots, we find that $c_{sd} \approx 0.85\sigma_{shot}$, indicating that the standard error presented in Fig. 3b is very close to the true time-dependent behavior of the standard deviation of the lattice response from a single shot.

As for obtaining a physical model, the standard deviation in Fig. 3 is crucial to characterizing the fluctuations in LLTO, since sample fluctuations will likely exhibit time-dependent behavior on scales associated with diffusion, charge transfer, or any dynamic processes directly influencing the measured quantity. In contrast, our probe would ideally not exhibit time-dependent behavior. Figure 3 also relates to the physics of our LLTO sample, in that from this analysis, we observe that the standard deviation relaxes faster than the motion of the lattice parameter itself, indicating a lattice stiffening behavior. We hope with these changes the connection between the standard deviation and observed correlations is made more clear.

**Rev. 1 Comment 4:** The correlation analysis in the second part is more powerful in this respect. It basically applies correlation methods that are commonly used in, e.g., x-ray photon correlation spectroscopy (XPCS), although the manuscript does not explicitly mention this. As these are well-established concepts, I find Fig. 4 somewhat inflated. It basically boils down to panel d showing the temporal autocorrelation. I do not see any value

in showing the two-time correlation functon(panel b) when the dynamics is temporally homogenous. Also, the value of the insets in panel a and the histogram in panel c is unclear.

We thank the Reviewer for bringing to our attention the close ties to methods in XPCS analysis. We understand that the two-time correlation employed in Fig. 4 is commonly calculated in XPCS to observe temporally inhomogeneous correlations. We will add corresponding citations to previous experiments employing a similar calculation.

**Added citations**: [Ragulskaya, Anastasia, et al. *J. Appl. Cryst.* **57.4** (2024)] [Chushkin, Yuriy, et al. *Phys. Rev. Lett.* **129.23** (2022): 238001] [Jain, Avni, et al. *J. Chem. Phys.* **157.18** (2022)]

**Edited main text in "Statistical analysis of lattice dynamics"**

Similar to calculations commonly employed in the analysis of X-ray photon correlation spectroscopy (XPCS) data, we represent correlations between $c_{ti}$ and $c_{tj}$ in a 2-D matrix, where each pixel is a unique mapping of i and j. This two-time correlation matrix is shown for its lower-half in Fig. 4b for all positive pump-probe time delays…

From the raw data presented in Fig. 2, it is not immediately obvious that correlations persist, and calculating a simple autocorrelation for each scan individually would not be robust as there are only 50 data points after time zero. Therefore, we computed the two-time correlation function across each of the 10 scans, under the assumption that there are no external changes to the sample or differing conditions between scans that skew the internal fluctuations (which is verifiable while looking at the data before time zero where the LLTO lattice reaches thermal equilibrium between pulse events). Therefore, showing the two-time correlation in Fig. 4b is necessary to demonstrate this key step of the calculation. We presented Fig. 4 in this manner to be transparent to the reader. On top of a visual representation representing homogeneity of temporal fluctuations, the 2-D matrix provides value from the standpoint of a pedagogical approach as this kind of analysis is not typically found in the majority of ultrafast literature. For this reason, the insets in panel a and the histogram are necessary in this regard. The insets in panel a show the level of correlation that is statistically significant near $t_i = t_j$. The histogram represents the distribution of all correlation coefficients, which is centered near zero with a positive skew due to the high correlations near $t_i = t_j$. This verifies that when $t_i$ is far from $t_j$ the correlation coefficients are normally distributed around zero indicating there are no other time-dependent correlations which may not be visibly apparent in the two-time correlation function. Therefore, from a transparency standpoint, the insets and histogram are necessary to convey the full meaning of the two-time correlation matrix, especially in the ultrafast pump-probe community where such analysis is uncommon.

We have adjusted the caption of Fig. 4 and corresponding text to be more scientifically accurate to hopefully enhance clarity in the communication of our analysis.

**Re-defined Fig. 4b,f** as "two-time correlation matrix"

**Re-defined Fig 4d,h** as "autocorrelation"

**Rev. 1 Comment 5:** While this kind of correlation analysis indeed provides some interesting insights into the underlying processes in the LLTO sample, I have strong concerns about the method's general applicability as it strongly depends on the presence of some degree of persistence or memory over a certain number of shots in the sample. The sample must switch between states or phases on the lab time scale (here, milliseconds) rather than on the time scale of the photo-response (here, microseconds but potentially picoseconds). In my view, this dramatically limits the application spectrum of the method (i) in terms of sample systems and (ii) to slow stochastic dynamics that can potentially be investigated by alternative, simpler methods.

We agree with the Reviewer that this method will only be applicable to systems that show a degree of persistence. The strength is that if such persistence exists, this method can distinguish this behavior from generic noise and thereby uncover phenomena that could be potentially overlooked in conventional pump-probe experiments. In fact, as we discovered correlations in our data, we thought deeply about the implications of this resulting in the present work. We believe that similar behavior was likely present in many past experiments where correlations were averaged out and thought of as noise. Despite that, we will of course be clear about this limitation in the manuscript and make the corresponding changes. To substantiate the breadth of phenomena where this method could potentially lead to new insights based on our current understanding, we surveyed literature again and we can now argue with specific examples that this limitation does not significantly narrow the scope of materials that exhibit stochastic processes. Here is a list of high impact studies across a wide range of systems that exhibit similar behavior:

- Nanoparticle/Quantum Dot 'blinking' phenomenon, where "on" and "off" fluorescence states persist over many seconds [Yuan, Gangcheng, et al. *ACS Nano*. **12.4** (2018): 3397-3405]
- Spontaneous spin switching [Weiss, M. A., et al. *Nat. Commun.* **14.1** (2023): 7651]
- Ultrafast nondeterministic erasure/writing of domain walls in 1T-$TaS_2$ [Zong, Alfred, et al. *Sci. Adv.* **4.10** (2018): 5501]
- Light-induced metastable states in $La_{2/3}Ca_{1/3}MnO_3$, $EuTe_4$ and $Ta_2NiSe_5$ and $PbTiO_3/SrTiO_3$ superlattices [Mcleod, Alexander S., et al. *Nat. Mater.* **19.4** (2020): 397-

404] [Zhang, Jingdi, et al. *Nat. Mater.* **15.9** (2016): 956-960] [Liu, Q. M., et al. *Nat. Commun.* **12.1** (2021): 2050] [Stoica, Vladimir A., et al. *Nat. Mater.* **23.10** (2024): 1394-1401].

- Chiral charge-density wave switching in $NbSe_2$ [Song, Xuan, et al. *Nat. Commun.* **13.1** (2022): 1843]

Many systems of interest in the materials science and condensed matter communities that exhibit metastable states are poorly understood, and often require extensive statistical analysis, and/or single-shot acquisition. In stroboscopic pump-probe experiments, statistics of the emergence and persistence of metastable states are difficult to obtain as photo-responses are averaged out. This leads to the next point, about the Reviewer's concern with the timescale of photo switching events. We agree that in the case of LLTO, we are observing lattice trajectory switching on a scale slower than the resolution provided by the probe. It is important to clarify that the millisecond timescale observed here is merely due to the repetition rate of the probe acquisition during this experiment (1 kHz). The control parameter in this experiment is laser pulses, not lab time. Essentially, choosing a different repetition rate would have changed the photo-induced trajectory switching timing in the lab time frame, but not the correlation length of 1,500 shots, which is the key finding of the study. Limitations arise in a scenario where the repetition rate is faster than the relaxation of the photoexcited lattice, which could yield false correlations as the system does not fully relax to equilibrium between events. For LLTO, the lattice fully relaxes within 1 millisecond as indicated by the uniformity of the lattice constant before time zero, so this problem is avoided.

Depending on the material process, employing our method would likely require careful consideration of the repetition rate and sampling size. There are many options to achieve higher repetition rates, and sometimes via pulse trains, such as:

- Mode-locked oscillators: nanoseconds
- Spatial light modulator (SLM) pulse shaping: picoseconds
- Monochromatized HHG: atto- to femto-seconds

These timescales will be useful to observe stochastic behavior in faster processes, such as screening effects, polaron formation, ultrafast magnetization, etc. Depending on the system of interest, access to its metastable-stochastic behavior may also depend heavily on the probe energy. Electronic excitations near the fermi-surface, optical fluorescence, or low-energy lattice modulations may be observable with low energy probes, whereas higher energy probes are able to access core-level electronic states or achieve high spatial resolution to observe behavior where stochastic process may have otherwise been obscured, such as in the case of LLTO. Considering the continued progress of high-signal

laser sources developed in recent years, our analysis is very versatile and has the potential to be generalized to many other pump-probe sources. In summary, we like to stress that the method is not limited to time scale and can equally be applied to faster and slower processes, and we will clarify this further in the main text.

**Main Text in the final paragraph of "Simulation and discussion"**

Our method is also not limited to stochastic processes observed on microsecond timescales. It can be equally applied to faster timescales if there is careful consideration of the choice of laser repetition rate and pulse averaging, which may suppress or enhance the observation of correlations.

**Rev. 1 Comment 6:** Most importantly, this correlation analysis will not work in the case where the dynamics show truly "random fluctuations" with "shot-to-shot variations" as it is advertised throughout the manuscript. All references provided as examples of a fluctuating sample response (Ref 11-13) fall into this category and could not be analyzed with the correlation method proposed by the authors.

We agree with the Reviewer that the correlation analysis employed here will be most useful in the case where persistence exists in the measured quantity. Of course, if there are no correlations, we would expect a flat line in the autocorrelation curve. However, whether the cited examples indeed fall into this category of zero correlations should not be taken for granted, and with our analysis method, we can now verify or falsify the assumptions of whether many photoinduced dynamics are truly random and independent from shot to shot.

Furthermore, observing zero correlations would still present useful knowledge because if the lattice response from each shot is a totally random and independent event, the shot-to-shot variation can be quantified trivially with the relationship $c_{sd} = \sigma_{scan}\sqrt{n}$, where *n* is the number of shots averaged in each datapoint in a given scan. Such variations may be related to the intrinsic properties of the sample of interest or may be a result of the fluctuations of the pump or probe laser power intensities. A distinction between these causes would have to be made on a case-by-case basis, which we have provided groundwork to do so in the first half of our analysis.

Given the context of our introduction, we see References 11—13 as appropriately cited, given that there was not a rigorous demonstration whether these systems are 'truly' random in their shot-to-shot response, therefore our analysis method could potentially be used to investigate correlative behavior in these systems.

We recognize though that our initial wording in this context can be misleading. We intend to convey our method mainly as a tool to uncover and characterize metastable states. Therefore, we will change relevant sentences to highlight the intended application and well-defined limitations of the method.

**Main text in "Introduction"**

Due to the necessity of repeated acquisitions, this method may only be applied to systems with a degree of persistence in the measured quantity. Such a constraint is relevant in the context of characterizing metastable states relating to lattice, charge, spin, and orbital degrees of freedom, where quasi-equilibrium conditions result in a non-uniform response from a light pulse.

**Rev. 1 Comment 7:** The authors also provide a "simulation" of the stochasticity in the data. However, this simulation is only an MC-like implementation using the statistical properties of the experimental data and does not contain any physical model. It is evident that such a model must recreate similar data with the same statistical signatures. I do not understand what can be learned from this model.

We thank the Reviewer for bringing up concerns about the simulation. The purpose of the simulation was to re-create the data in an identical manner as the experimental protocol to determine the origin of the observed trajectory switching. The simulation, although MC-like, verifies that the LLTO lattice after photoexcitation behaves as a Markov chain, where future outcomes are solely dependent on the grain's present state. On longer timescales (μs-ms), this insight suggests the presence of long-range atomic diffusion [Emery, J., et al. *J. Phys.: Condens. Matter* **14.3** (2001): 523], which follow a 'random-walk' between vacancies in a crystal lattice.

On the contrary, non-Markovian dynamics exhibit non-exponential autocorrelation dynamics. In a non-Markovian system, future outcomes of the grain dynamics would depend on its history, such as cases with periodic dynamics, or where a backflow of information from the grain environment results in time-lagged correlations [Breuer, Heinz-Peter, et al. *Phys. Rev. Lett.* **103.21** (2009): 210401] [Doerries, Timo J., et al. *J. Stat. Mech.: Theory Exp.* **2021.3** (2021): 033202]. Given our exponential autocorrelation decay, it suggests that stochastic behavior in LLTO is the result of atomic diffusion. We will make corresponding changes for clarification.

**Main text in "Simulation and discussion"**

Physically, $p_0$ represents the probability for a micro-grain of LLTO to randomly change its nonequilibrium lattice expansion and relaxation trajectories after the next pump shot. The close agreement between the simulated and experimental data, particularly the exponential autocorrelation curve (Fig. 4d,h) confirms that the observed lattice trajectory switching can be well described by a Markov chain, where future outcomes depend solely on the current state of the system rather than its accumulated history. Therefore, the photoexcited LLTO *c* lattice parameter behaves analogously to a 'random walk' or Brownian motion [Bhattacharya. "*Random walk, Brownian motion, and martingales*." Vol. 52. New York, NY, USA: *Springer* (2021)].

From an energetics perspective, the value of $p_0 = e^{-\frac{E_0}{k_B T}}$ implies an energy barrier of $E_0$ = 0.4 ± 0.1 eV, where $k_B$ is the Boltzmann constant and $T \approx 620$ K is the lattice temperature right after photoexcitation (see Supplementary Note 8 for an estimate of the photoinduced lattice temperature rise). We note that the value of $E_0$ is close to both theoretical and experimental values of the energy barrier for lithium ion migration in LLTO $E_b \approx 0.3$ -- 0.5 eV [Zhang, Lu, et al. *Nat. Commun.* **11.1** (2020): 3490][Woodahl, Clarisse, et al. *Nature Materials* **22.7** (2023): 848-852][Geng, H. X., et al. *J. Alloys Compd.* **481**.1-2 (2009): 555-558]. Hence, a potential scenario is that lithium ion displacement after each pump pulse results in a slight variation in the metastable lattice structure.

**Main text in "Statistical analysis of lattice dynamics"**

This statistical analysis hence gives a quantitative measure of how fast the dynamical correlation is lost as more photoexcitation events occur under repeated pump shots, which originates from persistence behavior of the micro-grain. Knowing this correlation length, we can now compute the single-shot distribution of the LLTO lattice parameter (see Supplementary Note 6 for details). Interestingly, we find that $c_{sd} \approx 0.85\sigma_{shot}$, which means that the statistics presented in Fig. 3 provide a close description of the possible distributions of lattice parameters from a single shot as a function of time.

The simulation also verifies the suggested lattice stiffening behavior. The only way to recreate the fast relaxation in the standard deviation compared to the average lattice motion from Fig. 3 is to set the initial photo-response length of the lattice inversely proportional to the relaxation time of our fit function, suggesting that Lithium hopping also plays a role in providing a local free energy minimum for the lattice to relax during the diffusion process. We also were able to fine tune the parameter $p_0$ in the simulation to match exactly the correlation decay seen in Fig. 4, which allows us to extract the energy barrier associated with the photo-induced trajectory switching. Although this simulation is purely statistical, it is very powerful for confirming the stochastic behavior of LLTO which is closely connected to

its physics. Finally, the benefit of using an abstract model allows us to recreate the observations without dependence on the microscopic parameters of LLTO, which means that we can use a similar approach to other light-induced stochastic trajectories and not limit ourselves to the LLTO system alone. To summarize, from the statistical model employed we have learned that a Markov chain is a sufficient model to describe trajectory switching, we have extracted the exact probability of trajectory switching due to a single shot ($p_0$), and we discovered the lattice stiffening behavior, all relevant in the context of lithium hopping in LLTO.

**Rev. 1 Comment 8:** At some points in the manuscript, the language is too exaggerated. The authors frequently use the term “hidden correlations”, while a simple autocorrelation is sufficient to unveil the correlations. The term “disordered fluctuations” is a pleonasm. I am also not convinced that the method presented deserves its own name (“nonequilibrium noise correlation spectroscopy”) as all experimental and statistical tools are well-established, and also their combination is not particularly original. The terminology is even scientifically inaccurate, as the “correlation” analysis can only be applied under quasi-equilibrium conditions. Otherwise, the Arrhenius assumption would not work. In line 230, the authors are “Inspired by the excellent fit of Eq. (1) to the averaged response in Fig. 3a”. However, this is a standard generic model with many fit parameters, which is able to fit almost any time-resolved data of this kind. “[...] probing stochastic processes” (line 27) on ultrafast time scales is certainly challenging but not puzzling and, thus, not a “conundrum.”

We thank the Reviewer for the feedback regarding our language and terminology. We agree there are instances where the language can be improved, and we will correct instances where the terminology is redundant or misleading specific to each Reviewer’s point.

I. The authors frequently use the term “hidden correlations”, while a simple autocorrelation is sufficient to unveil the correlations.

   **Changed title**: Photoinduced correlations in stochastic dynamics of a solid-state ionic conductor.

   **Removed**: “hidden correlations”, removed “hidden” in the introduction

II. The term “disordered fluctuations” is a pleonasm.

   **Removed term** “disordered fluctuations”

   **Introduction paragraph 2**: Due to the necessity of repeated acquisitions, this method may only be applied to systems with a degree of persistence in the measured quantity. Such a constraint is relevant in the context of characterizing metastable

states relating to lattice, charge, spin, and orbital degrees of freedom, where quasi-equilibrium conditions result in a non-uniform response from a light pulse.

III. I am also not convinced that the method presented deserves its own name ("nonequilibrium noise correlation spectroscopy") as all experimental and statistical tools are well-established, and also their combination is not particularly original.

To this point, we have observed closely related techniques using similar language to describe the methodology. Weiss et. al. [Weiss, M. A., et al. *Nat. Commun.* **14.1** (2023): 7651] discovers ultrafast spin switching using "Femtosecond Noise Correlation Spectroscopy." There also exist techniques such as "Diffuse Correlation Spectroscopy", and "Fluorescence Correlation Spectroscopy" both relevant in characterizing biological processes [Wang, Quan, et al. *NeuroImage* **298** (2024): 120793] [Yu, Lan, et al. *Front. Phys.* **9** (2021): 644450]. Our method differs in that we employ this analysis on pump-probe data where photoexcitation drives the system away from equilibrium, hence "nonequilibrium" refers to the material state after photoexcitation. Given the general applicability of this method, "Nonequilibrium Noise Correlation Spectroscopy" is an accurate concise description of the technique which allows for overlap with other fields.

IV. The terminology is even scientifically inaccurate, as the "correlation" analysis can only be applied under quasi-equilibrium conditions. Otherwise, the Arrhenius assumption would not work.

As for the quasi-equilibrium sample conditions, we are using this term as a more specific description of the "nonequilibrium" state after photoexcitation. LLTO is indeed in a quasi-equilibrium state, since the lattice relaxation observed is on the scale of microseconds, and the temperature is well defined at each point. Thus, it is valid to classify LLTO as in a quasi-equilibrium state even though it is further away from its complete equilibrium condition (pre-photoexcitation). This is further supported by faster time resolved measurements where initial charge transfer due to photoexcitation has fully recovered and energy is diffused into heat [Pham, Kim H., et al. *JACS.* **147.30** (2025): 26456-26467]. As demonstrated in Supplementary Note 7, we can in principle have a well-defined temperature of the system at any time delay.

V. In line 230, the authors are "Inspired by the excellent fit of Eq. (1) to the averaged response in Fig. 3a". However, this is a standard generic model with many fit parameters, which is able to fit almost any time-resolved data of this kind.

We agree to soften the language regarding our justification for employing the fit equation Eq. (1), however we chose to keep this model as the purpose of this is not to fit the time traces, rather, to characterize relationship between the fitting parameters. The main two are the lattice expansion immediately after photoexcitation $I_0$, and the relaxation time $\tau$. We were able to find the relationship between these two parameters through a purely statistical model which reveals crucial information about the physics of the system. It is also worth mentioning that the correlation analysis in Fig. 4 is independent of the fit function parameters and does not significantly skew results of the correlation length of LLTO. To summarize, we have demonstrated that phenomenological recreation of the dataset is sufficient to uncover the nature of the trajectory switching.

**Main text in "Simulation and discussion" replaced** "Inspired by the excellent fit of Eq. (1) to the averaged response in Fig. 3a…" with "Using the fit function Eq. (1) from the averaged response in Fig. 3a…"

VI. "[…] probing stochastic processes" (line 27) on ultrafast time scales is certainly challenging but not puzzling and, thus, not a "conundrum."
**Replaced**: "conundrum" with "challenge"

**Rev. 1 Comment 9:** In summary, the authors have not convincingly explained how their method can be applied to study photoinduced processes with "random fluctuations," which manifest in the data as "shot-to-shot variations" beyond simply detecting the existence of these fluctuations. The sample case presented is undoubtedly interesting, but it remains specific. I see substantial limitations of the application of the method in terms of X-ray sources, sample systems, and time scales of the dynamics, which directly limit the impact of the work. Major revision of the manuscript is required to address these aspects.

We again thank the Reviewer for the very helpful comments that have substantially improved the quality of our paper. To summarize, our method provides a broadly applicable framework for uncovering and characterizing stochastic and metastable processes in condensed matter systems by separating intrinsic sample fluctuations from source noise. While we acknowledge challenges in certain sources such as FELs and HHG, our approach is generalizable across many experimental platforms, especially with continued advances in laser stability, filtering algorithms, and high-repetition-rate probes. We have demonstrated that our approach provides physically meaningful insights into sample dynamics revealing

persistence and trajectory switching in LLTO that would otherwise be obscured. Importantly, our framework validates the Markovian behavior of the LLTO crystal lattice, while connecting to its underlying physics. By clarifying our terminology, refining definitions, and emphasizing limitations, we highlight that this is not a new statistical invention but a versatile application of established methods in a nonequilibrium pump–probe context, with direct relevance to a wide range of materials exhibiting metastable states.

## Reviewer #2

I have co-reviewed the manuscript with one of the listed reviewers.

We appreciate the effort to co-review and acknowledge Reviewer 2 for the contributions they made to the overall report.

## Reviewer #3

**Rev. 3 Comment 1:** The manuscript “Hidden correlations in stochastic photoinduced dynamics of a solid-state ionic conductor” presents a method termed “nonequilibrium noise correlation spectroscopy”, which describes a statistical framework for extracting shot-to-shot correlations in pump–probe experiments. Applied to the transition metal compound Li0.5La0.5TiO3 and using synchrotron-based pump-probe X-ray diffraction, the study reveals correlated lattice responses with a characteristic “correlation length” of approximately 1,500 laser shots, corresponding to a switching energy of ~0.4 eV.

The work is relevant as it introduces a new statistical approach to extract correlations between individual pump shots in a stroboscopic X-ray pump–probe experiment. Unlike conventional methods that average out fluctuations or focus on single delay times, this technique quantifies shot-to-shot variations using synchrotron measurements with high stability and photon flux. The work meets the standards expected for publication in a journal such as Nature Communication. The method is described comprehensively, such that it should be possible to reproduce the results following the sample preparation, experimental measurement and statistical analysis description.

The manuscript is of good quality, the fit parameters used in the equations and simulations are clearly explained, and the influence of possible external noise sources is addressed. As the work is sound and concerns an important question, we feel that it will be suitable for publication in Nature Communications if the authors address the following questions :

We thank the Reviewer for the accurate summary and overall positive feedback on the methodology and presentation of the findings. We will address the following concerns to enhance the clarity and details of the manuscript.

**Rev. 3 Comment 2:** The manuscript presents multiple, distinct time scales—microseconds, seconds, and minutes—that are somewhat confusing. In both the "Time-resolved X-ray diffraction" section and the caption of Fig. 2, the same experimental procedure is described, but the relationship between these time scales is not entirely clear from neither of them. Only later, when the correlation analysis in Fig. 4 is discussed, it is stated that 1 μs corresponds to 1000 pump shots. Is our understanding correct that Fig. 2c,d show delay times $t_{1}...{}_{59}$, each spaced by 1 μs, but that there are 1000 pump shots (1 s) between each consecutive delay time point $t_i$ and $t_{i+1}$? The authors should clarify this in the main text where the results of Fig. 2 are discussed, and revise the caption of Fig. 2 to better explain the connection between the various time scales. We suggest also labeling the axes in Fig. 2c,d more clearly (e.g. explicitly marking $t_i$ and noting n = 1000 shots), which would make the figure more intuitive and more clearly linked to the schematic in Fig. 2a.

We thank the Reviewer for seeking clarification regarding the explanation of the experimental protocol. We have modified the caption of Fig. 2 and clarified further in the main text to state how each time scale is related to each other, and which are relevant for the conclusions made.

**Main text**: "The repetition rate of the ultraviolet pump laser was 1 kHz, and the X-ray diffraction peak was measured at each pump-probe delay time ranging from -8 to 50 μs in 1 μs steps ($t\_{i=1,...,59}$). Diffraction intensities of *n* pump-probe pulse pairs were averaged for each $t_i$ for 1 second, meaning that each pixel in Fig. 2c is an average of *n* ≈ 1,000 pulse pairs."

**Main text**: Modified wording in Fig. 2 caption for clarity.

**Rev. 3 Comment 3:** The authors should add a few sentences explaining the possible physical origin of the correlations (length) observed between different delay times.

We thank the Reviewer for seeking further clarification on the interpretation of the extracted correlation length. The fact that we observe a finite correlation length is ultimately due to the

Markovian behavior of the photoinduced lattice trajectory switching, meaning that there is a likelihood $p_0$ that the lattice will change its photoexcited pathway given a pump pulse. Our interpretation is that this is due to atomic diffusion through the lattice, temporarily changing the metastable structure of LLTO. To make these points, we have made the corresponding changes in the main text.

**Main text in "Statistical analysis of lattice dynamics"**

This statistical analysis hence gives a quantitative measure of how fast the dynamical correlation is lost as more photoexcitation events occur under repeated pump shots, which originates from persistence behavior of the micro-grain. Knowing this correlation length, we can now compute the single-shot distribution of the LLTO lattice parameter (see Supplementary Note 6 for details). Interestingly, we find that $c_{sd} \approx 0.85\sigma_{shot}$, which means that the statistics presented in Fig. 3 provide a close description of the possible distributions of lattice parameters from a single shot as a function of time.

**Main text in "Simulation and discussion"**

Physically, $p_0$ represents the probability for a micro-grain of LLTO to randomly change its nonequilibrium lattice expansion and relaxation trajectories after the next pump shot. The close agreement between the simulated and experimental data, particularly the exponential autocorrelation curve (Fig. 4d,h) confirms that the observed lattice trajectory switching can be well described by a Markov chain, where future outcomes depend solely on the current state of the system rather than its accumulated history. Therefore, the photoexcited LLTO *c* lattice parameter behaves analogously to a 'random walk' or Brownian motion [Bhattacharya. "*Random walk, Brownian motion, and martingales*." Vol. 52. New York, NY, USA: *Springer* (2021)].

From an energetics perspective, the value of $p_0 = e^{-\frac{E_0}{k_B T}}$ implies an energy barrier of $E_0$ = 0.4 ± 0.1 eV, where $k_B$ is the Boltzmann constant and $T \approx$ 620 K is the lattice temperature right after photoexcitation (see Supplementary Note 8 for an estimate of the photoinduced lattice temperature rise). We note that the value of $E_0$ is close to both theoretical and experimental values of the energy barrier for lithium ion migration in LLTO $E_b \approx$ 0.3 -- 0.5 eV [Zhang, Lu, et al. *Nat. Commun.* **11.1** (2020): 3490][Woodahl, Clarisse, et al. *Nature Materials* **22.7** (2023): 848-852][Geng, H. X., et al. *J. Alloys Compd.* **481**.1-2 (2009): 555-558]. Hence, a potential scenario is that lithium ion displacement after each pump pulse results in a slight variation in the metastable lattice structure.

**Rev. 3 Comment 4:** It is stated that, because only one Bragg peak (not a ring) is observed, the signal must originate from a single grain. Was it explicitly verified that no ring appears in any of the individual shots of each scan? If so, how good is the shot-to-shot position stability of this Bragg peak?

We thank the Reviewer for bringing up concerns about the spatial resolution capabilities of the method. Indeed, no diffraction rings were observed in any of the raw data collected for the scans presented in this study (As observed in Fig. 1d, where a sharp well-defined Bragg peak is shown, such is the case for all points in this scan). However, it is worth mentioning that we did not measure single-shot data, rather the raw diffraction images are an average of 1,000 shots (or one time delay exposure). Therefore, we cannot verify the shot-to-shot position stability of the Bragg peak directly. But we have estimated through further statistical analysis that the shot-to-shot standard deviation is very similar to the scan-to-scan standard deviation calculated in Fig. 3b (Included in Supplementary Note 6). At the highest value of the standard deviation (0.008 Å), we would expect the Bragg peak angle to change by only 0.05º, which indicates that the shot-to-shot position stability is rather robust. Also, during the experiment we could deterministically scan the sample position finding areas with weaker rings but Bragg peaks emerging (i.e., hitting a single grain mostly but also beam size probably larger than that particular grain), areas with multiple Bragg peaks displaced in angle (i.e., hitting 2-3 larger grains), and finally areas with clearly only one set of strong diffraction peaks (i.e. hitting a single crystal with a size fitting well to the microfocused X-ray beam). Scanning across the sample was reproducible and we could purposely position the beam onto such an area of interest where we then conducted the experiment. During runs, we never intermittently observed any other diffraction pattern shape, and the patterns were stable throughout the entire experiment. This makes us highly confident that indeed we measured a single grain in a reproducible manner.

**Added "Supplementary Note 6: Shot variation of the *c* lattice parameter"**

As mentioned in the main text, the standard deviation calculation in Fig. 3b ($c_{sd}$) is proportional to the standard deviation as a measure of individual laser shots to the sample ($\sigma_{shot}$). This section will calculate $\sigma_{shot}$ while considering the correlation time obtained from the fit in Fig. 4d.

Since we do not have access to the lattice response after an individual pulse event, $c_{sd}$ is generally referred to as the standard error, where this quantity is calculated over a group of data that is already averaged over 1,000 shots. To extract the true standard deviation ($\sigma_{shot}$), we can use the relationship below where *n = 1,000* shots is the sample size.

$$\sigma_{scan}^2 = \frac{\sigma_{shot}^2}{n} \times \sum_{i,j=0}^{n} \rho_{i,j}$$

$\rho_{i,j}$ is the correlation function presented as the fit in Fig. 4d that arises from correlated pulse events. For $n = 1{,}000$ shots and $\xi = 1{,}500$ shots, we find that $c_{sd} \approx 0.85\sigma_{shot}$, indicating that the standard error presented in Fig. 3b is very close to the true time-dependent behavior of the standard deviation of the lattice response from a single shot.

**Added to Methods:**

The diffraction pattern was stable and reproducible throughout the experiment. By scanning across the sample, regions corresponding to well-defined Bragg peaks were located and selected for the measurements.

**Rev. 3 Comment 5:** How can the authors be sure that the signal between different shots is coming from the same grain?

We thank the Reviewer for the insightful question; we know from the SEM image in Fig. 1c, that the grains range in size around a few micrometers. Due to our X-ray spot size, we illuminate a relatively small number of grains such that individual Bragg peaks are observed. Essentially, a Bragg peak corresponding to the *c* axis was chosen and was not close to any other Bragg peaks (i.e., there are no other peaks on the camera at this position). Since we did not observe any large angle changes of this Bragg peak (see our previous related response), it is safe to assume that it is the same Bragg peak. Also, considering this scan happened over just a few minutes, a large change in azimuthal diffraction angle would have been observed. Should the signal between different shots come from different grains, it will be too coincidental that the peaks all fall into the seam area of the detector as the grains would have to have their crystal axes perfectly oriented in the same direction.

**Rev. 3 Comment 6:** Possibly related to point 4, in Supplementary Note 2 the authors mention using a long-term drift correction. Given the presence of several distinct time scales in this experiment, they should clarify what is meant by "long." Specifically, how much drift correction is needed for the pre-time-zero averages, and over what time frame and how does this relate to the different time scales (see question 1). Does the extracted "correlation length" agree with and without drift correction?

We thank the Reviewer for careful attention to detail regarding our explanation of the drift correction. By "long" we mean over the course of minutes. Since each data point is acquired over 1 second at 1 kHz, the dataset in Fig. 2 is about 10 minutes of lab time acquisition. Since

we are concerned with the shot-to-shot statistics of LLTO, the small drift over a few minutes does not change the 1,500-shot correlation length or extracted parameters. The drift correction scaled the *c* lattice parameter in a few scans down by a maximum of $1\times10^{-3}$ Å. A figure comparison is shown in Supplementary Note 2.

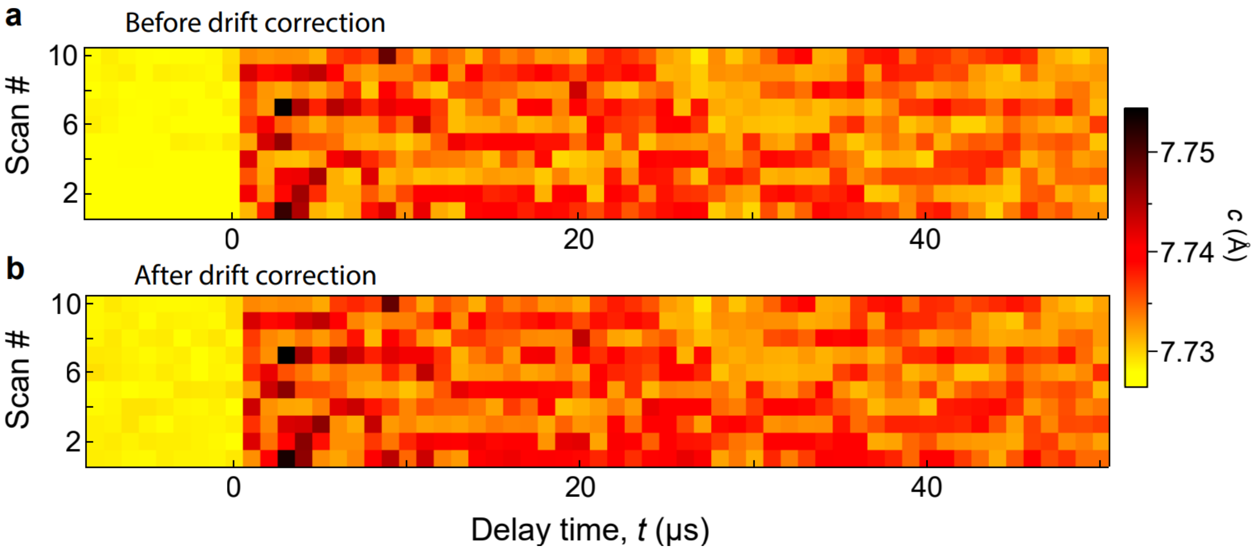


## Reviewer #4

I co-reviewed this manuscript with one of the reviewers who provided the listed reports. This is part of the Nature Communications initiative to facilitate training in peer review and to provide appropriate recognition for Early Career Researchers who co-review manuscripts.

We appreciate the effort to co-review and acknowledge Reviewer 4 for the contributions they made to the overall report. We appreciate the inclusion of early-career scientists and thank them for their time.

# Response to Reviewers for "*Photoinduced correlations in stochastic dynamics of a solid-state ionic conductor*"

February 11, 2026

We thank all referees for their careful review and constructive feedback, which have substantially helped us improve the quality of this work. Below, we provide a point-by-point response, where the referees' comments are shown in **black**, our responses are shown in **teal**, and manuscript amendments are shown in **red**.

## **Reviewer #1** (Remarks to the Author):

**Rev. 1 Comment 1**: I appreciate the effort the authors have put into revising the manuscript and providing an extensive rebuttal. The high quality of the synchrotron micro-diffraction data is unquestionable, and the observation of stochastic photoinduced pathways in LLTO remains both original and of scientific impact within the ultrafast science community. Figures 1 and 2 are exceptionally clear and effectively guide the reader through the experimental scheme and the observed phenomenology.

We thank the Reviewer for taking their time to read our revisions and for providing thorough and thoughtful comments. We appreciate the acknowledgement of the high data quality and figures.

**Rev. 1 Comment 2**: That said, even after revision, I find several aspects of the presentation stylistically inflated. Many of the core observations are conceptually straightforward, yet they are described in a lengthy and overly elaborate manner that does not improve clarity. This issue was not resolved in the revised version and, if anything, the rebuttal reinforced my impression that simple points are being presented with unnecessary complexity.

We thank the reviewer for commenting on the language and clarity. We agree that there is some stylistically inflated wording that can be removed. We identified several instances in the manuscript where explanations are unclear. To remove unnecessary complexity and improve clarity, we made several changes throughout the manuscript as listed under "changes" at the end of this comment.

The expanded discussion around the standard deviation is a good example. Although the rebuttal attempts to justify its importance, the manuscript itself never integrates this quantity into the physical discussion or uses it to advance the interpretation. As a result, it remains unclear why this analysis is needed or how it contributes to the main conclusions.

We agree with the Reviewer that the discussion around the standard deviation is overly complex and shrouds the reader from its purpose in the manuscript. The standard deviation is one step in the logical flow of the manuscript, and is crucial to the interpretation, as matching these dynamics to the simulation is what yields insight on the lattice stiffening behavior. We removed the unnecessary wording and provided a straightforward explanation for why the observation is crucial.

Similarly, my concerns about Fig. 4 remain. The extended two-time correlation matrix and its accompanying subfigures still feel disproportionate to the insight they provide, especially given the simulation's ineffectiveness (see following comment).

As for the presentation of Fig. 4, we respectfully disagree with changing its presentation. We would first like to acknowledge that the analysis may not be novel in other contexts such as XPCS, but utilizing this approach in a different context opens new scientific opportunities. Specifically, we aim to approach Fig. 4 as a guiding tool for the analysis, which is new to the ultrafast pump-probe community. Reviewer #1 states "*Figures 1 and 2 are exceptionally clear and effectively guide the reader through the experimental scheme and the observed phenomenology."* Although one can make an argument on the style of Fig. 4, we believe Fig. 4 follows the same philosophy as Fig. 1 and Fig. 2, effectively communicating the steps from calculating the 2D correlation matrix and conversion to the autocorrelation curve. The philosophy is echoed by Reviewer #3's comment, who stated, "*In addition, reviewer #1 raises some concerns regarding the presentation of the results which I support to a certain extent. But in my opinion, this is principally a matter of style and wording rather than content."* Finally, we stress that such a pedagogical approach helps us reach the broader readership of *Nature Communications*, especially those who are not experts in this type of analysis.

**Changes to improve clarity and reduce stylistic inflation:**

**Abstract**:

*(Pg. 2) Re-wrote from line 9*: "Leveraging the stability and high photon-flux of time-resolved X-ray micro-diffraction at a synchrotron, we employed established statistical tools to quantitatively characterize the stochastic behavior of the photoinduced dynamics in a solid-state electrolyte."

*(Pg. 2) Line 20*: replaced "a new strategy" with "insights"

**Introduction**:

*(Pg. 2) Line 25*: remove "rapid"

*(Pg. 2) Line 29*: remove "random" and "nondeterministic quasiparticle motions"

*(Pg. 3) Re-written from line 43*: "Here, we tackle this challenge in studying stochastic dynamics via a statistical approach to observe correlations between random events which reveal underlying physical properties of the material system. Although similar statistical analyses have been performed on dynamic chemical and biological flow systems [Yu, Lan, et al. *Front. Phys.* **9** (2021): 644450] [Wang, Quan, et al. *NeuroImage* **298** (2024): 120793] [Takazawa, Shuntaro, et al. *Phys. Rev. Res.* 5.4 (2023): L042019], our method focuses on the dynamic behavior of an individual microscopic grain and employs a stroboscopic data acquisition scheme as used in non-single shot pump-probe experiments. Due to the necessity of repeated data acquisitions, one requirement of this method is the use of a stable measuring probe and high measurement statistics to mitigate the averaging over several pulse events. The key to this method relies on the low noise level associated with…" "…photoinduced response. This method, with careful consideration of measurement parameters like repetition rate and data sampling, can be used to further characterize metastable states related to lattice, charge, spin, and orbital degrees of freedom where quasi-equilibrium conditions result in a non-uniform response from a light pulse."

*(Pg. 4) Lines 67 and 72*: remove "highly"

*(Pg. 4) Line 83*: removed “novel”

**Simulation and Discussion:**

*(Pg. 11) Line 290:* remove “hence”

*(Pg. 11-12) Re-written from line 310*: “Our results demonstrate a statistical method to track and characterize photoinduced dynamics that are not reproducible over the course of a stroboscopic measurement. We expect this technique to be applicable to a variety of pump-probe experiments, provided that stochastic behavior can be conclusively assigned to the system, rather than experimental uncertainties. With fine-tuning of experimental parameters regarding the pulse repetition rate, data binning, and use of a highly stable pump-probe setup, one can extract correlations in averaged data on a variety of timescales of interest.”

*(Pg. 12) Line 328*: replace “powerful” with “useful”

*(Pg. 11 & 12) Lines 307 and 328*: remove “hence”

*Remove*: Supplementary Note 6

**Changes regarding the standard deviation discussion:**

**Time-resolved X-ray micro-diffraction**:

*(Pg. 6) Line 154*: “This decrease in variation indicates that there is an underlying convergence of lattice motion at longer time delays compared to initial photoexcitation conditions, which suggests a correlation between the grain conditions in the short-lived photoexcited regime compared to the long-time thermal relaxation, which we will characterize in the following section.”

*(Pg. 6) Remove from line 156*: “For independent uncorrelated events, the relationship between these two quantities depends only on *n*, the number of shots at one delay time.”

*(Pg. 7) Line 162*: “Both observations are not typically observed in simple thermal expansion and relaxation models without the presence of a phase transition or mobile charges modulating the lattice dynamics. We will quantify both features and relate each to the underlying physics of the photoinduced dynamics.”

*(Pg. 7) Line 162*: remove “hence”

*(Pg. 9) Remove from line 236*: “Knowing this correlation length, we can now compute the single-shot distribution of the LLTO lattice parameter (see Supplementary Note 6 for details). Interestingly, we find that $c_{sd} \approx 0.85\sigma_{shot}$, which means that the statistics presented in Fig. 3 provide a close description of the possible distributions of lattice parameters from a single shot as a function of time.”

**Rev. 1 Comment 3**: Simulation

The value of the simulation remains unclear. As currently implemented, it is essentially tautological: the authors observe an exponential decay of correlations, simulate a generic process that is explicitly constructed to exhibit the same exponential decay, and then conclude that the simulation "reproduces" the experimental behavior. This does not provide additional physical insight, nor does it validate the proposed mechanism.

We thank the reviewer for carefully considering the purpose of our simulation. We agree that the motivation of the simulation should have been stated with a clearer objective, and the physical picture should have been described more clearly. However, we respectfully disagree that the simulation is tautological or yields no physical insight.

Like in other combined experiment/simulation studies, the goal of the simulation is to extract physical parameters of the system, which do in fact quantify microscopic phenomenon. By matching the average and standard deviation with the experiment, we can explicitly demonstrate the negative correlation between $I_0$ and $\tau$ while ruling out alternative scenarios involving changes in other parameters [see Eq. (1) in the manuscript] describing the evolution. This analysis specifically points to a physical picture where the crystalline lattice of LLTO stiffens at elevated temperatures, an important piece of insight as we quantify the structural dynamics of the ionic conductor.

Next, by understanding how our simulation reproduces the correlation lengths in the experiment, we can extract $p_0$, and in turn perform an estimate of the energy barrier between existing states. This corresponds to the energy barrier between the '*N*' possible trajectories following a photoexcitation event. These conclusions cannot be confidently drawn without the simulation.

Finally, based on the simulation result, we were able to connect these observations with the microscopic picture of lithium ion hopping, which we discuss in length in the "Simulation and discussion" section. Briefly, $p_0$ corresponds to the energy scale of Li hopping, and the $I_0$ — $\tau$ correlation provides a description of the free energy of the system in the nonequilibrium state.

In fact, the observed dynamics correspond more closely to a simple telegraph-noise (two-state switching) model with a characteristic lifetime rather than to a genuine random walk.

We also want to address the classification of our model. We thank the reviewer for pointing out that our simulation does resemble more of a telegraph-noise than a random walk, and we have removed mention the term 'random-walk'. However, there are not enough statistics accumulated in this work to claim the number of possible photoexcited states, nor where they lie in the photoexcited regime. As a comprehensive approach, we chose to model *N*-state switching, where the number of possible photoexcited states is not specified, but rather drawn from a distribution of possible states. The choice of number of possible states is not crucial to the correlation time nor the relationship between $I_0$ and $\tau$, therefore our interpretation does not change. Using the approach adopted considering Reviewer 1 Comment 2, we now think that drawing comparisons to these models does not provide new insight and is not necessary for the main conclusions. What is crucial is the characterization of the Markovian behavior of the *c* lattice parameter, which we establish in the manuscript.

I would encourage the authors either to substantially reframe the purpose of the simulation as purely illustrative or to develop a model that discriminates between alternative physical scenarios rather than reproducing the statistics by construction.

We again thank the Reviewer for taking their time to consider the value of our simulation. To clear any confusion regarding the purpose of the simulation, we strengthened the discussion by proposing questions that the simulation helps resolved at the start of "Simulation and discussion." We also made modifications as indicated below to enhance clarity. However, we respectfully disagree with reframing the purpose of the simulation, as it is not "purely illustrative." We use this simulation to explicitly extract parameters related to the photoexcited behavior of LLTO that otherwise would have been obscured.

**Changes**

**Statistical analysis of Lattice dynamics**:

*(Pg. 8) Line 189*: Removed "means that the *c*-axis parameter becomes more consistent in value at larger delay times"

*(Pg. 8) Added at the end of Line 194*: "This insight provided by reproducing the statistical signatures from the simulation demonstrates a nonlinear force-displacement relationship, confirming *c*-axis lattice stiffening in the thermally excited regime."

**Simulation and discussion**:

*(Pg. 10) Line 271*: Remove "Therefore, the photoexcited LLTO *c* lattice parameter behaves analogously to a 'random walk' or Brownian motion."

We revised the manuscript on page 10 by clearly stating the objective of the simulation before presenting the technical details, hence positioning the readers to understand the open questions addressed by the simulation.

*(Pg. 9) Line 241*: "Despite the insight provided by our analysis above, we need to explore further to extract the physical origin of the correlation in the stochastic lattice expansion and relaxation, as well as its connection to the underlying dynamics. To reveal the nature of the stochastic behavior, we…"

**Rev. 1 Comment 4**: Applicability of the Method

The applicability of the proposed method still appears overstated. Its usefulness hinges on long-lived shot-to-shot persistence over thousands of pump pulses, which is a highly unusual condition in ultrafast pump-probe experiments. As such, the method cannot reveal genuinely random fluctuations, e.g., those near a critical point or governed by fast, nondeterministic quasiparticle dynamics, despite the phrasing in the introduction suggesting otherwise.

We thank the Reviewer for raising concerns with our wording regarding the applicability of the method. We agree there should be further clarification on the purpose of our method. However, we respectfully disagree that the present application requires unusual conditions. Specifically, the Reviewer raises concerns about the "long-lived" persistence, which 1.) is indeed relevant 2.) can be addressed by experimental design.

**Relevance**: As suggested by the Reviewer and what may have been misleading from our manuscript, our goal is ***not*** to "reveal genuinely random fluctuations," i.e., proving the random nature of the fluctuations. Instead, we aim to demonstrate that pump-probe techniques can be

used to track the dynamics of systems that are not fully reproducible from pulse to pulse, from which, we can get insights into the microscopic physics as demonstrated in this study.

In the context of systems such as near the critical point of a phase transition, it can be misleading to think of correlations in a pure statistical, stationary sense. For example, in a highly ordered system, such as spins aligned in an Ising model, the statistical correlation of spin orientations is very high. However, upon a pump pulse, say we perturb several spins, then the fluctuations become highly randomized. Therefore, the pumped state can act differently from pulse-to-pulse, resulting in some finite but small correlation time. As the system passes through a second-order phase transition, it is typically the case that correlations of these fluctuations become longer in space and time, meaning that near $T_c$, one may see highly similar excited states between pulses. Hence, the scheme developed in our study can observe and quantify the correlated dynamics of the critical fluctuation via a temperature-dependent measurement, offering insights into a variety of phase transitions in quantum materials.

**Experimental design**: We agree that it can be challenging to access correlations that are lost on shorter time scales, say a few hundred shots, or even tens of shots, but the versatility of pump-probe data acquisition allows fine tuning of the integration time (data binning) and repetition rate. With the existence of high photon flux sources, we believe it to be very likely that a similar experiment can reveal faster correlation times, where the main constraint would be the signal-to-noise ratio as we approach the single shot limit.

Finally, we agree with the reviewer that the discussion around the applicability of this method in the introduction and discussion sections is inflated, so we adjusted the introduction to not overstate the potential applications of our method.

**<u>Changes</u>: (See changes from Rev. 1 Comment 2 to soften language)**

**Simulation and discussion**:

*(Pg. 12) Line 322*: replace “hold great potential” with “relevant”

*(Pg. 12) re-wrote from Line 328*: “This work provides a useful tool to characterize correlations in otherwise random dynamics that occur at the intrinsic timescale and length scale of electronic, ionic, and molecular motion.”

**Rev. 1 Comment 5**: In their rebuttal, the authors list several examples of metastable or switching phenomena from the literature. These systems do indeed exhibit persistence in various forms, but they are not integrated into the manuscript.

We thank the reviewer for comments on the relevance of related work. We cited the examples of metastable state/switching phenomena in the introduction of the manuscript that we believe can benefit from our analysis (see citations under “Changes” below).

In addition, they do not clearly demonstrate the specific type of long-lived, shot-correlated stochastic dynamics required for the present method. Some of the cited works probe dynamics that are directly observable in single-shot measurements or rely on deterministic photoinduced switching rather than hidden correlations.

We thank the reviewer for considering our examples and pointing out differences in the stochastic behavior. Here, we address the citations mentioned in our previous response letter:

- Ultrafast nondeterministic erasure/writing of domain walls in 1T-$TaS_2$ [Zong, Alfred, et al. *Sci. Adv.* **4.10** (2018): 5501]
    - Charge density wave (CDW) domains are switchable due to photoexcitation and are non-deterministic, but the probability can be tuned by fluence and temperature and persist until cooling. This is a system where shot-to-shot correlations likely do play a role as the pulse induced defects depend on the sample history.
- Light-induced metastable states in $La_{2/3}Ca_{1/3}MnO_3$, $Ta_2NiSe_5$ and $PbTiO_3/SrTiO_3$ [Mcleod, Alexander S., et al. *Nat. Mater.* **19.4** (2020): 397-404] [Zhang, Jingdi, et al. *Nat. Mater.* **15.9** (2016): 956-960] [Liu, Q. M., et al. *Nat. Commun.* **12.1** (2021): 2050] [Stoica, Vladimir A., et al. *Nat. Mater.* **23.10** (2024): 1394-1401]
    - These studies demonstrate a long-lived optically induced phases, which are observable over accumulations of single shots. This is an example of pumping a system that doesn't fully relax between the repetition rate of the laser. To observe shot-to-shot correlations of the accumulated excited state, our method can be tuned to observe potential correlations after the system fully relaxes.
- Chiral charge-density wave switching in $NbSe_2$ [Song, Xuan, et al. *Nat. Commun.* **13.1** (2022): 1843]
    - Chiral CDW switching is stochastic and persists over many milliseconds [Song, et al (2022) *Supplementary Figure 4*]. The probability of chiral CDW switching can be tuned by an external electric field. As an example, our method can be employed to observe correlations in the CDW out of equilibrium.

Concerning the references currently cited in the manuscript: The authors agreed that the persistence required was not reported or implied in these works. The claim that "it could still be the case" that they do so is speculative and unsupported.

While we agree that there is a degree of speculation and uncertainty regarding which specific systems will host correlations like what we observe in our study, although our reasoning is not unsupported. We assume the Reviewer is referring to references: [Wall 2018] [Zong 2021] [Perez-Salinas 2022] and [Disa 2023] (Ref. 14-17), reporting on $VO_2$, $DyTe_3$ & $LaTe_3$, $La_{0.5}Sr_{1.5}MnO_4$, and $YTiO_3$ respectively. We agree that the current context for these citations may not be fully appropriate as these works do not elucidate metastable behavior, therefore we moved these references to another part of the introduction. Regarding our wording however, we have not stated *"it could still be the case"* in the manuscript. Instead, we aim to focus on the citations listed above [Zong 2018] [McLeod 2020] [Zhang 2016] [Liu 2021] [Stoica 2024] [Song 2022] that correspond to pump-induced metastable states. We have also included two more appropriate citations: [Ilyas, Batyr, et al. *Nature* **636** (2024): 609-614] [Padma, Hari, et al. *Nature Materials* (2025): 1-8], which demonstrate persistent photoinduced metastable states in $FePS_3$ and $Sr_{14}Cu_{24}O_{41}$ respectively. As for the scientific case for potentially using our method to measure correlations in pump-induced metastable states, we stress that fluctuations at equilibrium will contribute to how the material system behaves in a nonequilibrium regime, and as an example, the photoinduced metastable magnetization in an otherwise antiferromagnetic $FePS_3$ is closely linked to the enhanced spin fluctuations near the Néel transition temperature [Ilyas 2024]. Therefore, it is not unfounded to claim our method may be used to investigate these systems, considering there is a finite correlation length for fluctuations in systems at equilibrium. Currently, the required shot-correlated persistence is experimentally demonstrated only in the LLTO system studied here; therefore, extension to other systems remains an open, testable question. Given this fact, we have softened

language regarding the applicability of our method but keep these references as potential applications of our method.

**<u>Changes</u>: (See all changes from Rev. 1 Comment 2 to soften language)**

*Moved References 14-17 to Line 56:* "In our experiment, we overcome these problems by utilizing the stability and high photon flux of a synchrotron-based time-resolved X-ray setup such that the experimentally observed signal variation is dominated by the intrinsic sample fluctuations in the photoinduced response [Wall, Simon, et al. *Science* **362** (2018): 572-576] [Zong, Alfred, et al. *Phys. Rev. Lett.* **127** (2021): 227401] [Perez-Salinas, Daniel, et al. *Nat. Commun.* **13.1** (2022): 238] and [Disa, A. S., et al. *Nature* **617** (2023): 73-78].

*Added citations in line 59*: [Mcleod, Alexander S., et al. *Nat. Mater.* **19.4** (2020): 397-404] [Zhang, Jingdi, et al. *Nat. Mater.* **15.9** (2016): 956-960] [Liu, Q. M., et al. *Nat. Commun.* **12.1** (2021): 2050] [Stoica, Vladimir A., et al. *Nat. Mater.* **23.10** (2024): 1394-1401] [Ilyas, Batyr, et al. *Nature* **636** (2024): 609-614] [Padma, Hari, et al. *Nature Materials* (2025): 1-8].

We excluded citations mentioned in the previous response on spontaneous random processes: [Yuan, Gangcheng, et al. *ACS Nano.* **12.4** (2018): 3397-3405] [Weiss, M. A., et al. *Nat. Commun.* **14.1** (2023): 7651].

*(Pg. 3) Re-written in introduction Line 56*: "This method, with careful consideration of measurement parameters like repetition rate and data sampling, can be used to further characterize metastable states related to lattice, charge, spin, and orbital degrees of freedom where quasi-equilibrium conditions result in a non-uniform response from a light pulse."

**Rev. 1 Comment 6**: Relation to XPCS

I appreciate that the revised manuscript now situates the method more explicitly within the context of correlation analysis techniques developed for XPCS. However, I would caution against drawing too close an analogy. A central strength of XPCS is its interference-based sensitivity, which allows extremely small structural fluctuations to be detected over relatively large illuminated volumes. The present method, by contrast, relies on micro-focused single-grain diffraction to achieve sufficient local sensitivity for observing stochastic switching. While this is well-suited to the current synchrotron experiment, it also introduces practical constraints, particularly in FEL settings, where achieving micron-scale focusing with adequate stability is technically challenging. Moreover, the high fluence of tightly focused FEL pulses raises significant concerns about radiation damage, which may preclude the repeated, persistent probing of a single micrograin that this method requires. Thus, the approach is complementary to XPCS but should not be presented as a streamlined substitute; its applicability remains inherently limited by the need for highly localized, low-damage probes and exceptional stability.

We thank the Reviewer for providing context on our method and its comparisons to XPCS and the use of FEL sources, and we appreciate the acknowledgement that our method is well suited in the current context. We agree with the Reviewer's comment on the strength of XPCS on speckle-based experiments, and its ability to access extremely small discontinuities over a large volume holds a significant advantage in this regard. We aim to make the distinction here as in the

case of this experiment, we require micro-focusing allowed by the synchrotron. We also agree that our approach is complementary to XPCS, as explicitly stated in the conclusion, and we did not intend to claim our method as a substitute.

As for the practical constraints of our method, we agree that small focusing, sample damage, and beam stability are all limitations worth careful consideration when designing an experiment such as this. However, these limitations are not impractical at state-of-the-art FEL sources given the rapidly improving shot-to-shot stability, flux attenuation, and micro-focusing techniques over the past several years. For example, LCLS, PAL-XFEL, EuXFEL are all able to focus X-rays below 1 μm at FWHM, while keeping relatively high stability and mitigating damage using beam filters and attenuators [Walter, Peter, et al. J. Synchrotron Radiat. 29.4 (2022): 957-968] [Ma, Rory, et al. Sci. Rep. (2025)] [Liu, Shan, et al. Synchrotron Radiat. News. 38.2 (2025): 11-16]. FELs such as these still hold benefits such as pump-probe capabilities, variable repetition rate, electronic pulse-delay control, stability monitoring, and focal control needed to realize similar experiments. Although we believe our method to be applicable to FELs, we did not make arguments for this in the manuscript, as it is not the goal of this study to present this work in an FEL context. We do agree with the reviewer however on the legitimate constraints and challenges, so we have expanded the discussion that clarifies these limitations as elucidated by the Reviewer.

**Changes:**

*(Pg. 12) Line 320*: "While XPCS can probe extremely small discontinuities over a large volume, our method differs in that a highly localized, low damage probe is necessary to access micro-scale physics."

***(From Rev. 1 Comment 2.)*** *On the limitations of our method*:

*(Pg. 3) Line 45*: "Although similar statistical analyses have been performed on dynamic chemical and biological flow systems [Yu, Lan, et al. *Front. Phys.* **9** (2021): 644450] [Wang, Quan, et al. *NeuroImage* **298** (2024): 120793] [Takazawa, Shuntaro, et al. *Phys. Rev. Res.* 5.4 (2023): L042019, our method focuses on the dynamic behavior of an individual microscopic grain and employs a stroboscopic data acquisition scheme as used in non-single shot pump-probe experiments. Due to the necessity of repeated data acquisitions, one requirement of this method is the use of a stable measuring probe and high measurement statistics to mitigate the averaging over several pulse events. The key to this method relies on the low noise level associated with…"

*(Pg. 12) Line 314*: "With fine-tuning of experimental parameters regarding the pulse repetition rate, data binning, and use of a highly stable pump-probe setup, correlations can be extracted from averaged data on a variety of timescales of interest."

**Rev. 1 Comment 7**: In summary, while the experimental data are of high quality and the observation of stochastic photoinduced pathways is genuinely interesting, the manuscript in its current form does not meet the standards for publication in Nature Communications. The scope and applicability of the method are overstated, key arguments remain speculative or insufficiently justified, and several aspects of the presentation obscure rather than clarify the underlying physics. Substantial revision would be required to address these issues. I therefore recommend major revision before the manuscript can be considered for publication.

We thank the reviewer for their thoughtful and constructive feedback, and we appreciate the interest in the observations. We believe that with the current changes, that we have refined and

made clear the scope of the manuscript, strengthened the physical interpretation, and improved clarity throughout the manuscript. By removing language throughout the manuscript that overstates novelty and generality, we reframed this method as a statistical approach to tracking photoinduced dynamics that demonstrate correlative behavior under stroboscopic pump-probe conditions. We also clarified the purpose of the simulation while removing unnecessary complexity of the corresponding text. Finally, we refined the applicability of the method along with its challenges in the conclusion to provide accurate context of this work relative to XPCS. With all these changes, we now believe that the manuscript is now ready for publication in *Nature Communications*.

## **Reviewer #3** (Remarks to the Author):

**Rev. 3 Comment 1**: The authors have thoroughly addressed the questions raised by us and the other reviewers, and in the revised manuscript provide additional information that improves the overall clarity.

We thank the Reviewer for carefully reviewing our revised manuscript and pointing out the improved clarity.

**Rev. 3 Comment 2**: One remaining point concerns our previously raised question 5 regarding long-term drifts. The authors explain the origin and explicitly stated in their response that the drift correction is $1\times10^{-3}$ Å. Given that this value is small and not visible in the before and after correction plot comparison, the authors should also include it in Supplementary Section 2.

We thank the reviewer for noticing this point about the small long-term drift of the micro-grain lattice constant, we have explicitly stated this correction value in Supplementary Note 2.

**Changes**:

*In Supplementary Note 2, Line 789*: ($\approx1\times10^{-3}$ Å)…

**Rev. 3 Comment 3**: Apart from this minor addition, we believe the manuscript is now suitable for publication in Nature Communications.

We thank the reviewer for their consideration and support for publication in *Nature Communications*.

**Rev. 3 Comment 4**: In addition, I have been asked to comment on the concerns of reviewer #1 from the current review round. Reviewer #1 also acknowledges the high quality of the synchrotron micro-diffraction data, as well as the originality and potential impact of the results and the technique for the ultrafast condensed matter physics community.

We thank both Reviewers for the acknowledgement of our data quality and originality of the work.

**Rev. 3 Comment 5**: In addition, reviewer #1 raises some concerns regarding the presentation of the results which I support to a certain extent. But in my opinion, this is principally a matter of style

and wording rather than content. Therefore, I still feel that the manuscript should be considered for publication. I suggest minor revisions where the authors should simplify and streamline the discussion and generally adapt / tone down the phrasing in the spirit of reviewer #1's comments. Finally, I support reviewer #1's request that also challenges in the applicability of the method are discussed more openly, e.g., concerning beam stability and sample damage at X-ray FELs.

We thank the Reviewer for offering their viewpoint on concerns regarding stylistic choices of the wording and figures. We have made revisions in context and wording at specific instances where Reviewer 1 points out a lack of clarity and unnecessary complexities. We also toned down the language throughout the manuscript, including places Reviewer 1 did not specifically point out as to not overstate the novelty and generality. These changes, in our opinion, have substantially improved the clarity of the presentation while making the work appeal to the broad readership of *Nature Communications*. These changes, however, do not shift the central finding or undercut the microscopic physical insights presented in the manuscript.

**Rev. 3 Comment 6**: With those revisions implemented I trust that the manuscript will meet the standards for publication in Nature Communications.

We thank the Reviewer again for their consideration.

## **Reviewer #4** (Remarks to the Author):

**Rev. 4 Comment 1**: I co-reviewed this manuscript with one of the reviewers who provided the listed reports. This is part of the Nature Communications initiative to facilitate training in peer review and to provide appropriate recognition for Early Career Researchers who co-review manuscripts.

We thank the Reviewer for their careful evaluation of the revised manuscript.
